# Supplementary material for: Asynchrony and functional diversity couple herbivore community dynamics to host plant diversity
Source: Nat Commun. 2026 Jan 15;17:762. doi: 10.1038/s41467-025-67990-0 (PMC12819501; doi:10.1038/s41467-025-67990-0)
Supplement: Supplementary file 1 — Supplementary Infomation [file 41467_2025_67990_MOESM1_ESM.pdf]

Supplementary Information

Wang et al.

Table of contents:

Supplementary sections
    Supplementary Methods
    Supplementary Note 1
Supplementary figures
    Figures 1-9
Supplementary tables
    Tables 1-27

Supplementary Methods

Alternative calculation for temporal stability and population stability

To further eliminate the potential bias caused by rare and potentially vagrant species (e.g., extremely low stability), we also utilized an alternative stability metric from Kvålseth et al.[1]

$$\text{stability} = \frac{\sqrt{\sigma^2+\mu^2}}{\sigma^2} \quad \text{(S1)}$$

The plot level-average population stability of tree growth and herbivores were calculated as the sum of all species' temporal stability indices (applying formula S1).

Sensitive analysis

To evaluate the role of herbivore abundance and richness in the bottom-up process, we substituted herbivore MPD with herbivore abundance or richness in separate alternative models for sensitive analysis. While we assumed that herbivore MPD best captures diversity effects because it combines abundance-weighted differences in not only species richness, but phylogenetic (and thereby functional) differentiation among species[2], models using herbivore abundance or richness may be helpful in evaluating the contribution of overall herbivore density and the mere number of species, respectively. We applied the same path model structure selection procedure as in the model where MPD was included, only replacing MPD with abundance/richness (see methods in the main text).

We ran additional sensitivity analyses for the path models in which we assessed the influence of monoculture plots and rare species (n<5), respectively. Specifically, to assess how the effects of monocultures influence the overall results (as in monocultures tree growth asynchrony is zero by definition), we excluded all monoculture data and fitted the path models of herbivore MPD for overall, specialist, and generalist herbivores. To exclude potential effects caused by rare and potentially vagrant species, an alternative stability metric (stability following Kvålseth et al[1].) was included in all linear models and SEMs for overall herbivores, generalists, and specialists.

Supplementary Note 1

(Please note: These findings were based on the additional analysis, which was described in the Supplementary Methods above.)

The overall patterns were similar but direct effects of tree species richness on herbivore abundance/richness and population stability were kept in the model (model variant 3, Fig. 1c) when herbivore MPD was replaced by herbivore abundance or richness (Figs S1-S2; Tables S2-S7). Moreover, the effects of tree species richness

were also largely indirect by influencing herbivore asynchrony, population stability, and herbivore abundance/richness (Figs S1-S2; Tables S2-S7) via tree functional diversity as well as asynchrony and population stability of tree growth (Figs. 2, 3, S1; Tables S2-S4). Our findings remained highly consistent when an alternative stability metric (stability following Kvålseth et al[1].) was used in our models (Fig. S3d-f, S4-S6; Tables S16-S22) and all rare species were included (Fig. S7; Tables S23-24).

Moreover, exclusion of monocultures (for which asynchrony is zero by default) expectedly led to weaker effects for some of the observed relationships, but the main pathways connecting tree species richness with herbivore stability remained strong and significant (Fig. S8; Tables S25-S27). When non-significant pathways were included, effects of tree asynchrony effects on all herbivores and of tree population stability on generalists were more prominent, but characterized by higher uncertainty (Fig. S9).

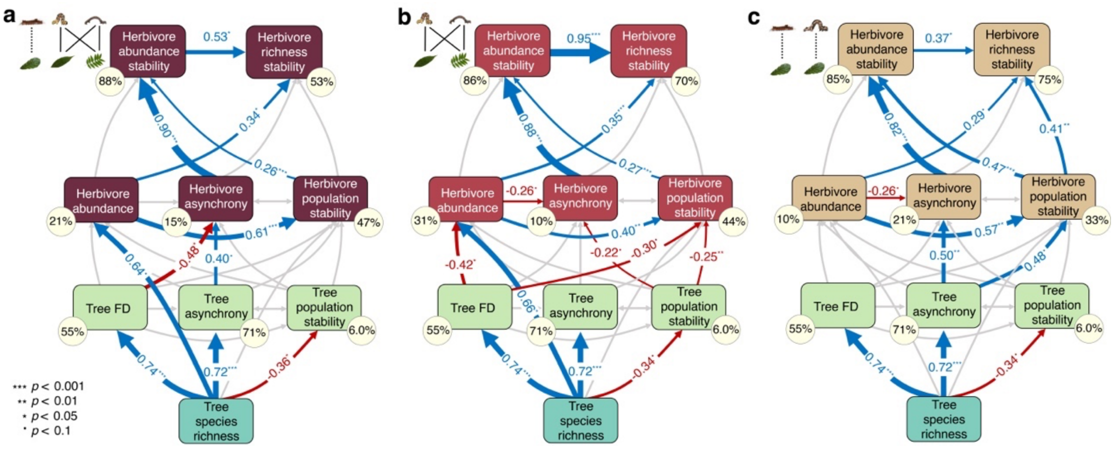

Fig. S1 Effects of tree diversity on herbivore community stability via bottom-up regulation using abundance instead of (mean phylogenetic diversity) MPD and stability based on the inverse of the coefficient of variation based on path model results. Potential effects of tree species richness (green rectangle), tree functional diversity (FD), species asynchrony and population stability (light green rectangles) on community stability of herbivore abundance and richness through herbivore abundance, species asynchrony and population stability for **a** overall herbivores (χ^2 =8.47, DF =9 , P =0.487; dark red rectangles), **b** generalist herbivores (χ^2 =7.73, DF =9, P =0.562; red rectangles), and **c** specialist herbivores (χ^2 =6.25, DF =9, P =0.715; light brown rectangles) based on path model results (see Tables S2-S4 for full results). Blue arrows indicate positive effects, red arrows show negative effects ($p \leq 0.1$), grey arrows show non-significant pathways ($p > 0.1$). Arrow width was scaled by the standardized path coefficients. The proportion of variance (R^2) are shown in yellow circles. Note that tree species richness, population stability, abundance stability and richness stability of herbivores were log-transformed. Stability measures are based on the inverse of the coefficient of variation (eqn. 3). Significance levels: p < 0.1 (·), p < 0.05 (*), p < 0.01 (**), p < 0.001 (***). Statistical tests were two-sided, and no adjustments were made for multiple comparisons.

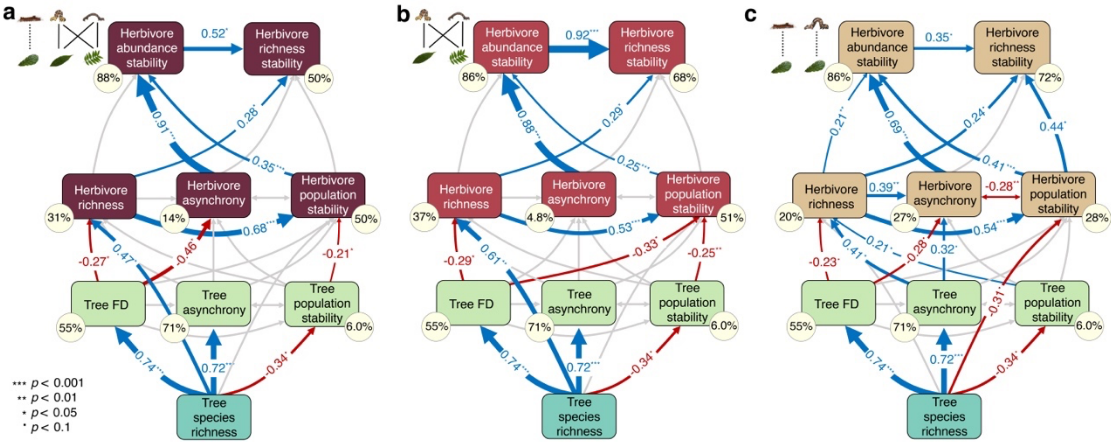

Fig. S2 Effects of tree diversity on herbivore community stability via bottom-up regulation using richness instead of (mean phylogenetic diversity) MPD and stability based on the inverse of the coefficient of variation based on path model results. Potential effects of tree species richness (green rectangle), tree functional diversity (FD), species asynchrony and population stability on community stability of herbivore abundance and richness through herbivore richness, species asynchrony and population stability for **a** overall herbivores (χ^2 =6.73, DF =9, P = 0.665; dark red rectangles), **b** generalist herbivores (χ^2 =7.17, DF =9, P =0.620; red rectangles), and **c** specialist herbivores (χ^2 =4.24 , DF =9, P =0.895; light brown rectangles) based on path model results (see Tables S5-S7 for full results). Blue arrows indicate positive effects, red arrows show negative effects ($p \leq 0.1$), grey arrows show non-significant pathways ($p > 0.1$). Arrow width was scaled by the standardized path coefficients. The proportion of variance (R^2) are shown in yellow circles. Note that tree species richness, population stability, abundance stability and richness stability of herbivores were log-transformed. Stability measures are based on **the inverse of the coefficient of variation** (eqn. 3). Significance levels: p < 0.1 (·), p < 0.05 (*), p < 0.01 (**), p < 0.001 (***). Statistical tests were two-sided, and no adjustments were made for multiple comparisons.

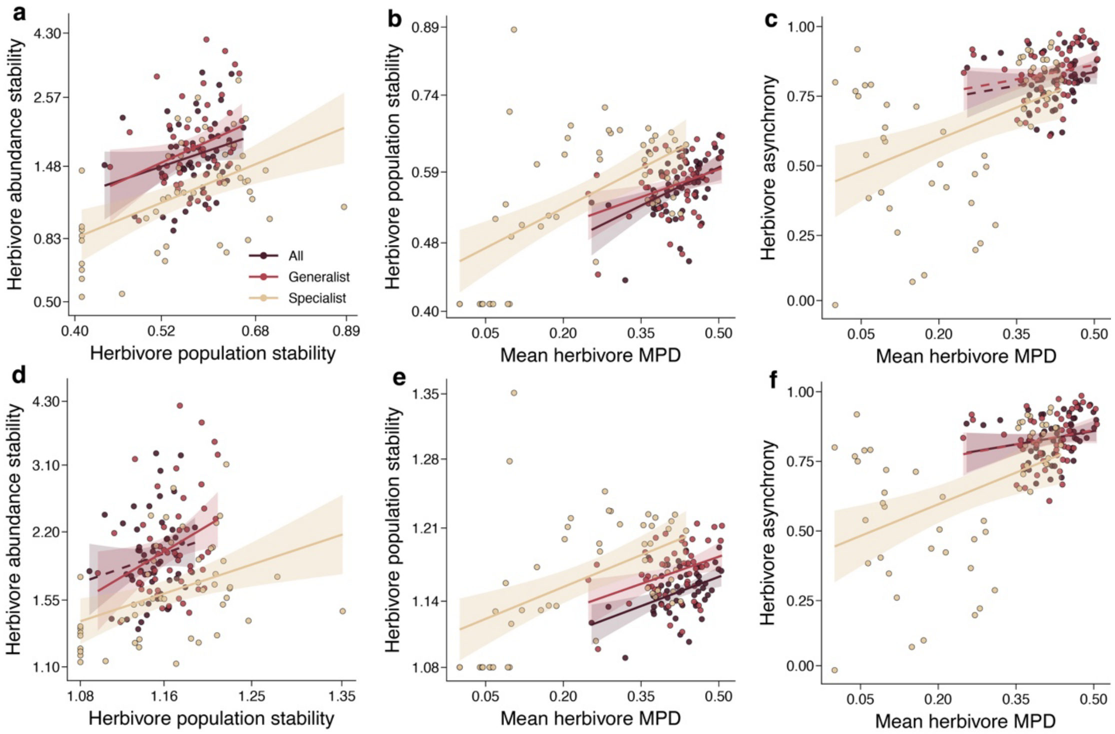

Fig. S3 Bivariate relationships between herbivore stability, asynchrony, population stability, mean phylogenetic diversity (MPD) based on linear model results. 'All' includes the entire herbivore community (generalists and specialists) analyzed together (All: light brown, generalist: red, specialist: dark red). Lines are linear regression model fits of the relationships between herbivore abundance stability and **a** herbivore population stability. Relationships between herbivore MPD and **b** herbivore abundance stability and **c** herbivore asynchrony. Stability metrics are calculated based on stability based on the inverse of the coefficient of variation (a-c) and stability following Kvålseth et al[1]. (d-f) (see Methods). Regression lines (with 95% confidence bands) show significant (solid lines, $p \leq 0.05$) or non-significant (dashed lines, $p > 0.05$). Note that population stability, abundance stability and richness stability of herbivores were log-transformed.

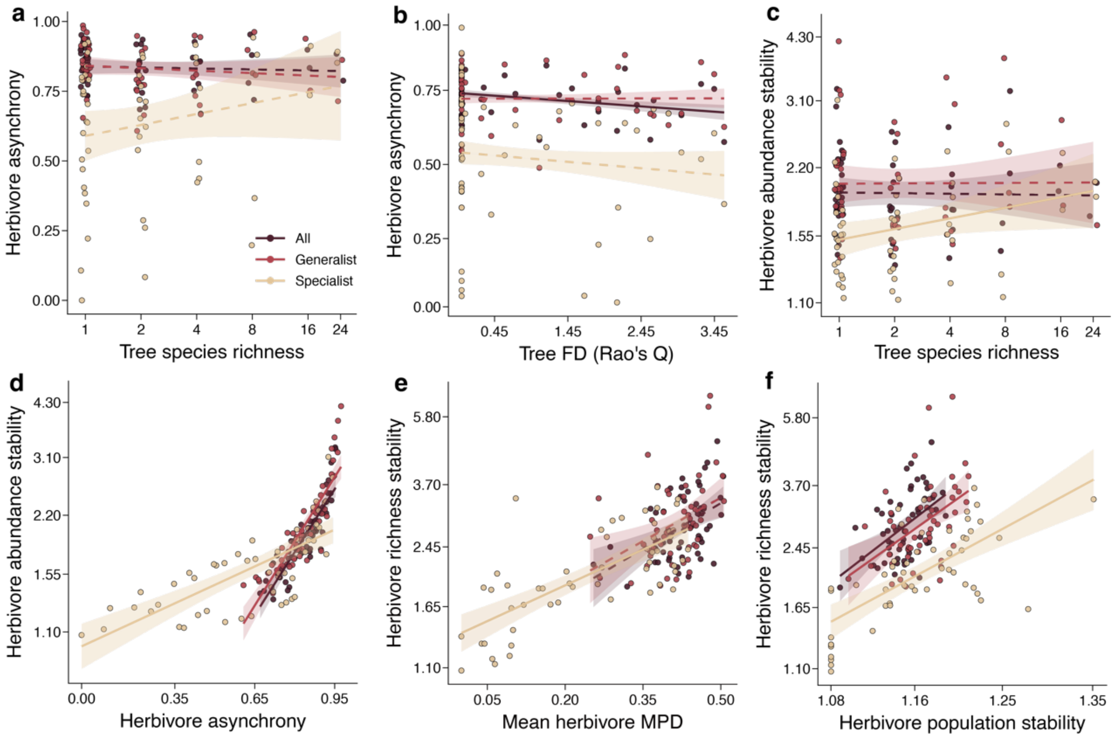

Fig. S4 Bivariate relationships between herbivore stability, asynchrony, population stability, mean phylogenetic diversity (MPD) and tree diversity using stability following Kvålseth et al[1] based on linear model results. 'All' includes the entire herbivore community (generalists and specialists) analyzed together (All: light brown, generalist: red, specialist: dark red). Lines are linear regression model fits of the relationships between herbivore asynchrony and **a** tree species richness and **b** tree functional diversity. Relationships between herbivore abundance stability and **c** tree species richness and **d** herbivore asynchrony. Relationships between herbivore richness stability and **e** herbivore MPD and **f** herbivore population stability. Regression lines (with 95% confidence bands) show significant (solid lines, $p \leq 0.05$) or non-significant (dashed lines, $p > 0.05$). Note that tree species richness, population stability, abundance stability and richness stability of herbivores were log-transformed. Stability measures are following Kvålseth et al[1]. (eqn. S1).

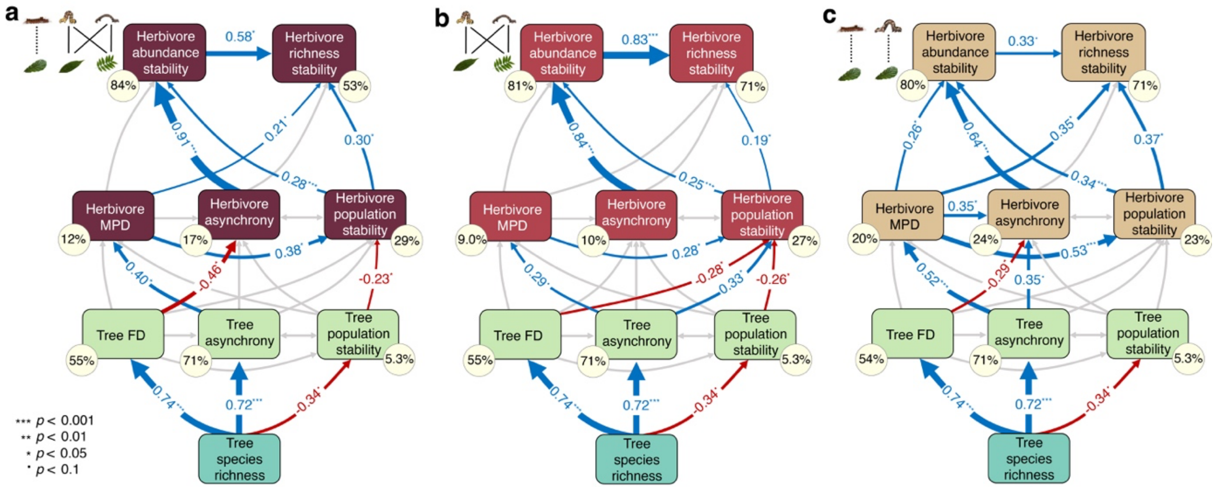

Fig. S5 Effects of tree diversity on herbivore community stability via bottom-up regulation based on path model results using stability following Kvålseth et al[1]. Potential effects of tree species richness (green rectangle), tree functional diversity (FD), species asynchrony and population stability (light green rectangles) on community stability of herbivore abundance and richness through herbivore abundance weighted phylogenetic diversity (MPD), species asynchrony and population stability for **a** overall herbivores (χ^2 =9.38 , DF =11, P =0.587; dark red rectangles), **b** generalist herbivores (χ^2 =12.13, DF =11, P =0.354; red rectangles), and **c** specialist herbivores (χ^2 =13.80 , DF =11, P =0.244; light brown rectangles) based on path model results (see Tables S20-S22 for full results). Blue arrows indicate positive effects, red arrows show negative effects ($p \leq 0.1$), grey arrows show non-significant pathways ($p > 0.1$). Arrow width was scaled by the standardized path coefficients. The proportion of variance (R^2) are shown in yellow circles. Note that tree species richness, population stability, abundance stability and richness stability of herbivores were log-transformed. Stability measures are following Kvålseth et al[1]. (eqn. S1). Significance levels: $p < 0.1$ (·), $p < 0.05$ (*), $p < 0.01$ (**), $p < 0.001$ (***). Statistical tests were two-sided, and no adjustments were made for multiple comparisons.

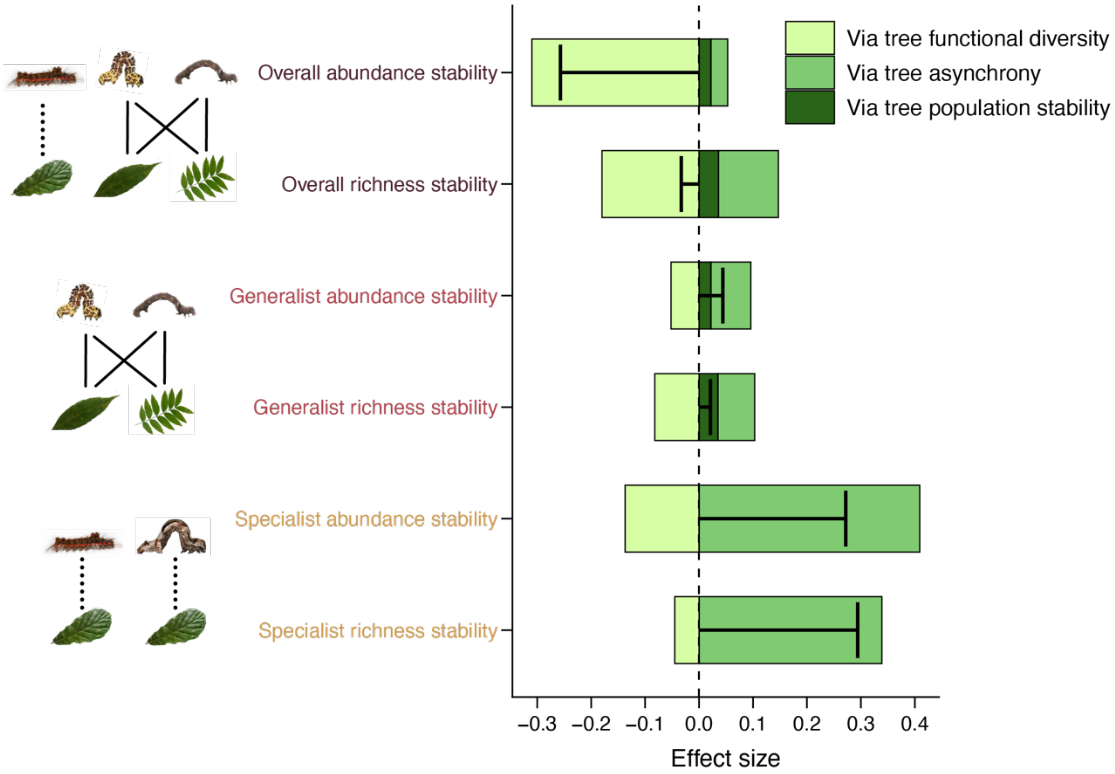

Fig. S6 Effects of tree species richness on herbivore community stability using stability based on path model results following Kvålseth et al[1]. Bars show summed effects of tree species richness on the abundance and richness stability of all, generalist and specialist herbivores, respectively. Effect sizes were calculated by summing indirect effects of tree species richness via tree functional diversity, tree asynchrony, tree population stability, herbivore mean phylogenetic diversity, herbivore asynchrony and herbivore population stability. The different colors show effects of tree species on herbivore stability via tree functional diversity (light green), tree asynchrony (green), and tree population stability (dark green), respectively. Effect sizes were calculated as the product of standardized path coefficients connecting each predictor with herbivore components, summed over the individual predictors of each component for positive and negative effects on herbivore stability metrics, respectively. Black T-shaped lines indicate the total effects of tree species richness on herbivore stability metrics. Note that the path coefficient based on the models with log-transformed variables (see above). Stability measures are following Kvålseth et al[1]. (eqn. S1). Source data are provided as a Source Data file.

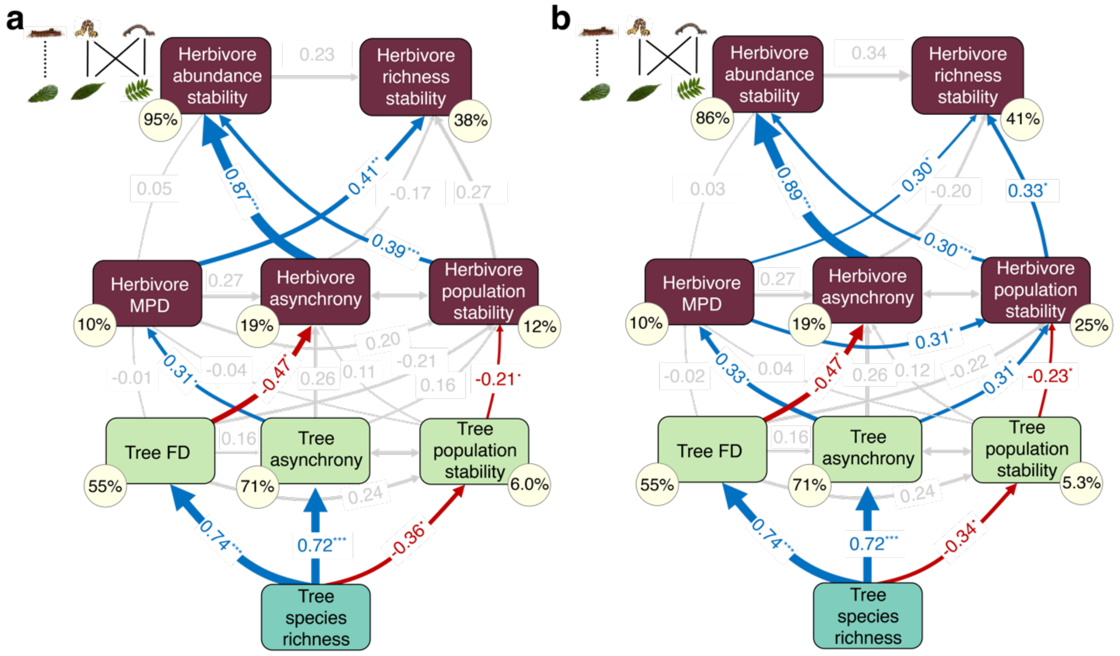

Fig. S7 Sensitivity analysis of path models for effects of tree diversity on herbivore community stability via bottom-up regulation including all herbivores. Potential effects of tree species richness (green rectangle), tree functional diversity (FD), species asynchrony and population stability (light green rectangles) on community stability of herbivore abundance and richness through mean herbivore abundance weighted phylogenetic diversity (MPD), species asynchrony and population stability for **a** overall herbivores using stability based on the inverse of the coefficient of variation (χ^2 =5.65 , DF = 11, P =0.0.890; dark red rectangles), **b** overall herbivores using stability following Kvålseth et al[1]. (χ^2 =11.19 , DF = 11, P =0.468; dark red rectangles) based on path model results (see Tables S23-S24 for full results). Blue arrows indicate positive effects, red arrows show negative effects ($p \leq 0.1$), grey arrows show non-significant pathways ($p > 0.1$). Arrow width was scaled by the standardized path coefficients. The proportion of variance (R^2) are shown in yellow circles. Note that tree species richness, population stability, abundance stability and richness stability of herbivores were log-transformed. Significance levels: p < 0.1 (·), p < 0.05 (*), p < 0.01 (**), p < 0.001 (***). Statistical tests were two-sided, and no adjustments were made for multiple comparisons.

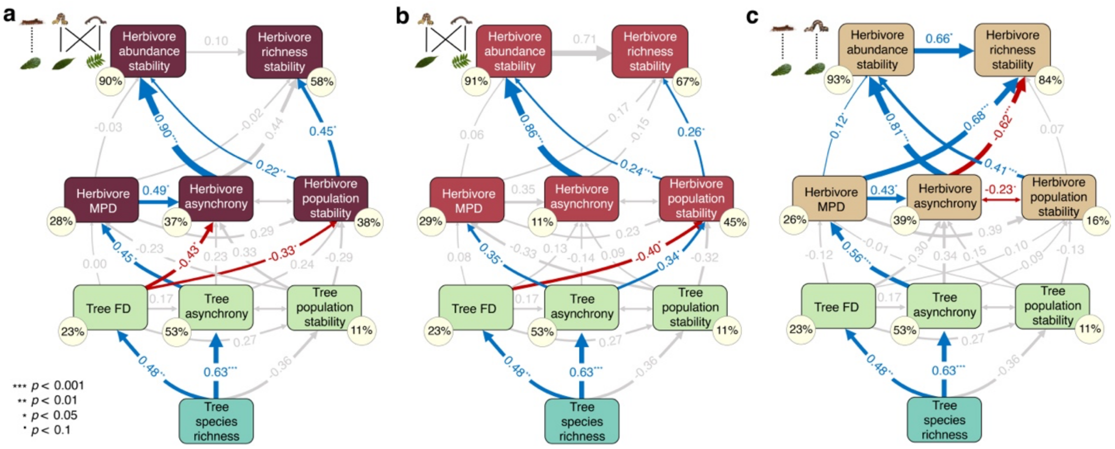

Fig. S8 Sensitivity analysis of path models for effects of tree diversity on herbivore community stability via bottom-up regulation using stability based on the inverse of the coefficient of variation and excluding monocultures. Potential effects of tree species richness (green rectangle), tree functional diversity (FD), species asynchrony and population stability (light green rectangles) on community stability of herbivore abundance and richness through herbivore abundance weighted phylogenetic diversity (MPD), species asynchrony and population stability for **a** overall herbivores (χ^2 =12.32 , DF = 11, P =0.340; dark red rectangles), **b** generalist herbivores (χ^2 = 13.89, DF =11 , P =0.239; red rectangles), and **c** specialist herbivores (χ^2 =9.08 , DF =11 , P =0.615; light brown rectangles) based on path model results (see Tables S25-S27 for full results). Blue arrows indicate positive effects, red arrows show negative effects ($p \leq 0.1$), grey arrows show non-significant pathways ($p > 0.1$). Arrow width was scaled by the standardized path coefficients. The proportion of variance (R^2) are shown in yellow circles. Note that tree species richness, population stability, abundance stability and richness stability of herbivores were log-transformed. Stability measures are based on the inverse of the coefficient of variation (eqn. 3). Significance levels: p < 0.1 (·), p < 0.05 (*), p < 0.01 (**), p < 0.001 (***). Statistical tests were two-sided, and no adjustments were made for multiple comparisons.

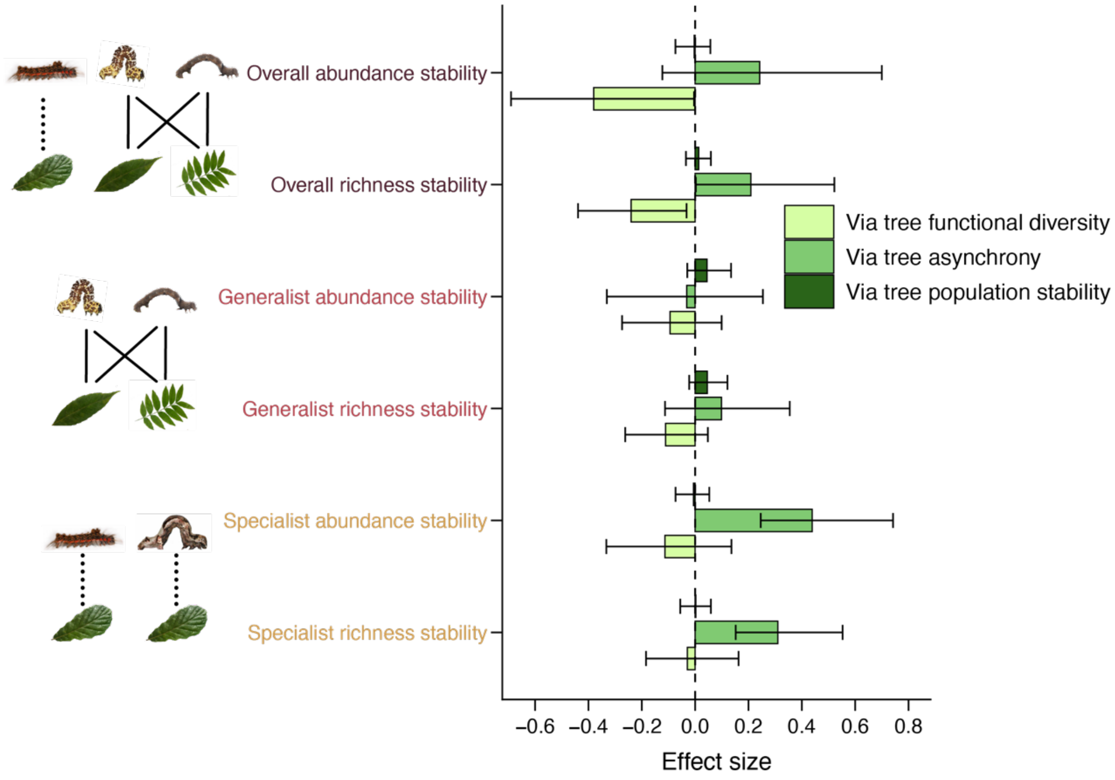

Fig. S9 Bootstrapped effects of tree species richness on herbivore richness stability based on path model results including all pathways (significant and non-significant). Bars show summed effects of tree species richness on the abundance and richness stability of all, generalist and specialist herbivores, respectively. Error bars indicate 95% bootstrap confidence intervals. Effect sizes were calculated by summing indirect effects of tree species richness via tree functional diversity, tree asynchrony, tree population stability, herbivore mean phylogenetic diversity, herbivore asynchrony, herbivore population stability, and herbivore abundance stability. The different colors show effects of tree species on herbivore stability via tree functional diversity (light green), tree asynchrony (green), and tree population stability (dark green), respectively. Effect sizes were calculated as the product of standardized path coefficients connecting each predictor with herbivore components, summed over the individual predictors of each component for positive and negative effects on herbivore stability metrics, respectively. Note that tree species richness, population stability, abundance stability and richness stability of herbivores were log-transformed. Stability measures are based on the inverse of the coefficient of variation (eqn. 3). Source data are provided as a Source Data file.

Table S1 Summary results of model fit statistics for the model selection procedure. Four potential models were considered during the path analysis selection procedure, each incorporating herbivore MPD, herbivore abundance, or herbivore richness, respectively. The fit statistics of the selected model are highlighted in bold. Stability measures are based on the inverse of the coefficient of variation (eqn. 3). Statistical tests were two-sided, and no adjustments were made for multiple comparisons.

**Herbivore MPD**	Model 1	Model 2	Model 3	**Model 4**
AIC	1110.63	1102.87	1100.97	**1097.55**
χ^2	0.00	4.25	6.34	**8.92**
p(χ^2)/ bootstrapped	NA	0.6430/0.581	0.609/0.579	**0.629/0.618**
DF	0.00	6.00	8.00	**11.00**
CFI	1.00	1.00	1.00	**1.00**
SRMR	0.000	0.012	0.018	**0.019**
RMSEA	0.00(90% CI: 0.00-0.00)	0.07 (90% CI: 0.00-0.15)	0.00 (90% CI: 0.00-0.14)	**0.00 (90% CI: 0.00-0.12)**
p(RMSEA)	NA	0.711	0.693	**0.725**

**Abundance**	Model 1	Model 2	**Model 3**	Model 4
AIC	1089.07	1082.32	**1080.54**	1081.75
χ^2	0.00	5.24	**7.46**	14.67
p(χ^2)/bootstrapped	NA	0.513/0.481	**0.488/0.476**	0.198/0.243
DF	0.00	6.00	**8.00**	11.00
CFI	1.00	1.00	**1.00**	0.99
SRMR	0.000	0.016	**0.020**	0.032
RMSEA	0.00(90% CI: 0.00-0.00)	0.00 (90% CI: 0.00-0.17)	**0.00 (90% CI: 0.00-0.15)**	0.08 (90% CI: 0.00-0.18)
p(RMSEA)	NA	0.593	**0.582**	0.294

**Richness**	Model 1	Model 2	**Model 3**	Model 4
AIC	1079.94	1072.41	**1070.62**	1070.52
χ^2	0.00	4.46	**6.68**	10.57
p(χ^2)/bootstrapped	NA	0.614/0.548	**0.572/0.589**	0.392/0.380
DF	0.00	6.00	**8.00**	11.00
CFI	1.00	1.00	**1.00**	0.99
SRMR	0.000	0.015	**0.024**	0.031
RMSEA	0.00(90% CI: 0.00-0.00)	0.00 (90% CI: 0.00-0.15)	**0.00 (90% CI: 0.00-0.14)**	0.03 (90% CI: 0.00-0.16)
p(RMSEA)	NA	0.685	**0.659**	0.500

Table S2. Path model output for tree and herbivore dynamics on overall herbivore community stability (herbivore MPD was replaced by abundance, stability measures are based on the inverse of the coefficient of variation). Statistical tests were two-sided, and no adjustments were made for multiple comparisons.

**Model**	
Estimator	Maximum likelihood
Number of observations	52
RMSEA	0.00 (90% CI: 0.00-0.15)
Chi-square	8.47
Degrees of freedom	9
P(Chi-square)/bootstrapped	0.487/0.476

Regressions

Response~Predictor	Estimate	SE	z	P	Stand. Est.
**Herbivore richness stability~**					
Herbivore population stability	0.18	0.17	1.01	0.314	0.18
Herbivore asynchrony	-0.01	0.30	-0.03	0.980	-0.01
Herbivore abundance	0.34	0.16	2.12	0.034	0.34
Herbivore abundance stability	0.53	0.32	1.59	0.095	0.53
**Herbivore abundance stability~**					
Herbivore population stability	0.33	0.07	4.48	0.000	0.33
Herbivore asynchrony	0.90	0.06	14.89	0.000	0.90
Herbivore abundance	-0.06	0.08	-0.75	0.454	-0.06
**Herbivore population stability ~**					
Tree population stability	-0.13	0.09	-1.43	0.154	-0.13
Tree asynchrony	0.26	0.24	1.10	0.271	0.26
Tree species richness	-0.06	0.25	-0.24	0.808	-0.06
Tree FD	-0.20	0.19	-1.09	0.275	-0.20
Herbivore abundance	0.61	0.16	3.73	0.000	0.61
**Herbivore asynchrony ~**					
Tree population stability	0.05	0.15	0.31	0.754	0.05
Tree asynchrony	0.40	0.22	1.79	0.074	0.40
Tree FD (Rao's Q)	-0.48	0.21	-2.21	0.027	-0.48
Herbivore abundance	-0.18	0.13	-1.38	0.169	-0.18
**Herbivore abundance ~**					
Tree population stability	-0.13	0.12	-1.12	0.264	-0.13
Tree asynchrony	-0.15	0.40	-0.36	0.716	-0.15
Tree species richness	0.64	0.42	1.53	0.089	0.64
Tree FD (Rao's Q)	-0.20	0.16	-1.23	0.220	-0.20
**Tree population stability ~**					

Tree species richness	-0.36	0.17	-2.16	0.031	-0.36
Tree FD (Rao's Q)	0.24	0.20	1.23	0.219	0.24
**Tree FD (Rao's Q) ~**					
Tree species richness	0.74	0.08	9.58	0.000	0.74
**Tree asynchrony ~**					
Tree species richness	0.72	0.21	3.45	0.001	0.72
Tree FD (Rao's Q)	0.16	0.20	0.80	0.422	0.16

Covariances	Estimate	SE	z	P	Stand. Est.
Herbivore population stability~~					
Herbivore asynchrony	0.02	0.09	0.23	0.816	0.03
Tree population stability~~					
Tree asynchrony	-0.02	0.04	-0.52	0.603	-0.04

Variances

Variable	Estimate	SE	z	P	Stand. Est.
Tree species richness	0.98	0.20	4.84	0.000	1.00
Herbivore population stability	0.52	0.11	4.70	0.000	0.53
Herbivore asynchrony	0.84	0.13	6.36	0.000	0.85
Tree FD (Rao's Q)	0.44	0.12	3.72	0.000	0.45
Herbivore abundance	0.78	0.19	4.17	0.000	0.79
Tree population stability	0.92	0.20	4.52	0.000	0.94
Tree asynchrony	0.28	0.09	3.07	0.002	0.29
Herbivore richness stability	0.47	0.06	7.78	0.000	0.47
Herbivore abundance stability	0.12	0.02	5.87	0.000	0.12

R^2

Variable	Estimate
Herbivore population stability	0.47
Herbivore asynchrony	0.15
Tree FD (Rao's Q)	0.55
Herbivore abundance	0.21
Tree population stability	0.06
Tree asynchrony	0.71
Herbivore richness stability	0.53
Herbivore abundance stability	0.88

Table S3. Path model output for tree and herbivore dynamics on generalist herbivore community stability (herbivore MPD was replaced by abundance, stability measures are based on the inverse of the coefficient of variation). Statistical tests were two-sided, and no adjustments were made for multiple comparisons.

**Model**	
Estimator	Maximum likelihood
Number of observations	52
RMSEA	0.00 (90% CI: 0.00-0.14)
Chi-square	7.73
Degrees of freedom	9
P(Chi-square)/bootstrapped	0.562/0.586

Regressions

Response~Predictor	Estimate	SE	z	P	Stand. Est.
**Herbivore richness stability~**					
Herbivore population stability	-0.01	0.10	-0.07	0.946	-0.01
Herbivore asynchrony	-0.22	0.20	-1.10	0.272	-0.22
Herbivore abundance	0.35	0.10	3.42	0.001	0.36
Herbivore abundance stability	0.95	0.23	4.15	0.000	0.95
**Herbivore abundance stability~**					
Herbivore population stability	0.27	0.07	4.10	0.000	0.27
Herbivore asynchrony	0.88	0.08	10.72	0.000	0.88
Herbivore abundance	0.01	0.07	0.08	0.938	0.01
**Herbivore population stability ~**					
Tree population stability	-0.25	0.09	-2.81	0.005	-0.25
Tree asynchrony	0.02	0.20	0.09	0.930	0.02
Tree species richness	0.32	0.26	1.20	0.232	0.32
Tree FD	-0.30	0.17	-1.77	0.078	-0.30
Herbivore abundance	0.40	0.13	3.03	0.002	0.40
**Herbivore asynchrony ~**					
Tree population stability	-0.22	0.13	-1.65	0.098	-0.22
Tree asynchrony	-0.05	0.21	-0.22	0.830	-0.05
Tree FD (Rao's Q)	-0.04	0.19	-0.23	0.816	-0.04
Herbivore abundance	-0.26	0.15	-1.76	0.078	-0.26
**Herbivore abundance ~**					
Tree population stability	-0.14	0.14	-1.02	0.308	-0.14
Tree asynchrony	0.09	0.29	0.31	0.755	0.09
Tree species richness	0.66	0.29	2.29	0.022	0.66
Tree FD (Rao's Q)	-0.42	0.18	-2.37	0.018	-0.42
**Tree population stability ~**					

Tree species richness	-0.36	0.18	-1.98	0.048	-0.36
Tree FD (Rao's Q)	0.24	0.21	1.13	0.258	0.24
**Tree FD (Rao's Q) ~**					
Tree species richness	0.74	0.07	10.42	0.000	0.74
**Tree asynchrony ~**					
Tree species richness	0.72	0.20	3.52	0.000	0.72
Tree FD (Rao's Q)	0.16	0.20	0.81	0.416	0.16

Covariances	Estimate	SE	z	P	Stand. Est.
Herbivore population stability~~					
Herbivore asynchrony	0.10	0.09	1.09	0.277	0.14
Tree population stability~~					
Tree asynchrony	-0.02	0.04	-0.53	0.593	-0.04

Variances

Variable	Estimate	SE	z	P	Stand. Est.
Tree species richness	0.98	0.21	4.71	0.000	1.00
Herbivore population stability	0.55	0.11	4.84	0.000	0.56
Herbivore asynchrony	0.88	0.18	4.88	0.000	0.90
Tree FD (Rao's Q)	0.44	0.12	3.66	0.000	0.45
Herbivore abundance	0.68	0.13	5.21	0.000	0.69
Tree population stability	0.92	0.22	4.28	0.000	0.94
Tree asynchrony	0.28	0.09	3.06	0.002	0.29
Herbivore richness stability	0.29	0.05	5.62	0.000	0.30
Herbivore abundance stability	0.14	0.04	3.86	0.000	0.14

R^2

Variable	Estimate
Herbivore population stability	0.44
Herbivore asynchrony	0.10
Tree FD (Rao's Q)	0.55
Herbivore abundance	0.31
Tree population stability	0.06
Tree asynchrony	0.71
Herbivore richness stability	0.70
Herbivore abundance stability	0.86

Table S4. Path model output for tree and herbivore dynamics on specialist herbivore community stability (herbivore MPD was replaced by abundance, stability measures are based on the inverse of the coefficient of variation. Statistical tests were two-sided, and no adjustments were made for multiple comparisons.

**Model**	
Estimator	Maximum likelihood
Number of observations	52
RMSEA	0.00 (90% CI: 0.00-0.12)
Chi-square	6.25
Degrees of freedom	9
P(Chi-square)/bootstrapped	0.715/0.602

Regressions

Response~Predictor	Estimate	SE	z	P	Stand. Est.
**Herbivore richness stability~**					
Herbivore population stability	0.41	0.16	2.52	0.010	0.41
Herbivore asynchrony	0.16	0.18	0.89	0.375	0.16
Herbivore abundance	0.29	0.20	1.45	0.098	0.29
Herbivore abundance stability	0.37	0.21	1.77	0.047	0.37
**Herbivore abundance stability~**					
Herbivore population stability	0.47	0.08	5.68	0.000	0.46
Herbivore asynchrony	0.82	0.08	10.15	0.000	0.82
Herbivore abundance	0.11	0.09	1.21	0.227	0.11
**Herbivore population stability ~**					
Tree population stability	-0.02	0.10	-0.22	0.825	-0.02
Tree asynchrony	0.48	0.25	1.91	0.056	0.48
Tree species richness	-0.47	0.29	-1.62	0.106	-0.46
Tree FD	0.04	0.17	0.20	0.839	0.04
Herbivore abundance	0.57	0.21	2.69	0.007	0.57
**Herbivore asynchrony ~**					
Tree population stability	0.05	0.14	0.34	0.735	0.05
Tree asynchrony	0.50	0.16	3.13	0.002	0.50
Tree FD (Rao's Q)	-0.27	0.18	-1.51	0.130	-0.27
Herbivore abundance	-0.26	0.14	-1.88	0.061	-0.26
**Herbivore abundance ~**					
Tree population stability	-0.10	0.14	-0.69	0.490	-0.10
Tree asynchrony	-0.37	0.46	-0.80	0.424	-0.37
Tree species richness	0.46	0.50	0.92	0.356	0.46
Tree FD (Rao's Q)	0.09	0.18	0.52	0.602	0.09
**Tree population stability ~**					

Tree species richness	-0.36	0.19	-1.95	0.051	-0.36
Tree FD (Rao's Q)	0.24	0.21	1.15	0.252	0.24
**Tree FD (Rao's Q) ~**					
Tree species richness	0.74	0.07	10.07	0.000	0.74
**Tree asynchrony ~**					
Tree species richness	0.72	0.22	3.34	0.001	0.72
Tree FD (Rao's Q)	0.16	0.21	0.76	0.447	0.16

Covariances	Estimate	SE	z	P	Stand. Est.
Herbivore population stability~~					
Herbivore asynchrony	0.03	0.10	0.25	0.799	0.04
Tree population stability~~					
Tree asynchrony	-0.02	0.04	-0.54	0.588	-0.04

Variances

Variable	Estimate	SE	z	P	Stand. Est.
Tree species richness	0.98	0.21	4.79	0.000	1.00
Herbivore population stability	0.66	0.19	3.56	0.000	0.67
Herbivore asynchrony	0.78	0.16	4.87	0.000	0.79
Tree FD (Rao's Q)	0.44	0.11	3.99	0.000	0.45
Herbivore abundance	0.88	0.29	3.08	0.002	0.90
Tree population stability	0.92	0.22	4.21	0.000	0.94
Tree asynchrony	0.28	0.09	3.01	0.003	0.29
Herbivore richness stability	0.25	0.04	5.95	0.000	0.25
Herbivore abundance stability	0.15	0.03	4.79	0.000	0.15

R^2

Variable	Estimate
Herbivore population stability	0.33
Herbivore asynchrony	0.21
Tree FD (Rao's Q)	0.55
Herbivore abundance	0.10
Tree population stability	0.06
Tree asynchrony	0.71
Herbivore richness stability	0.75
Herbivore abundance stability	0.85

Table S5. Path model output for tree and herbivore dynamics on overall herbivore community stability (herbivore MPD was replaced by richness, stability measures are based on the inverse of the coefficient of variation). Statistical tests were two-sided, and no adjustments were made for multiple comparisons.

**Model**	
Estimator	Maximum likelihood
Number of observations	52
RMSEA	0.00 (90% CI: 0.00-0.13)
Chi-square	6.73
Degrees of freedom	9
P(Chi-square)/bootstrapped	0.665/0.610

Regressions

Response~Predictor	Estimate	SE	z	P	Stand. Est.
**Herbivore richness stability~**					
Herbivore population stability	0.20	0.18	1.12	0.265	0.20
Herbivore asynchrony	-0.11	0.31	-0.36	0.719	-0.11
Herbivore richness	0.28	0.15	1.86	0.042	0.28
Herbivore abundance stability	0.52	0.33	1.59	0.038	0.52
**Herbivore abundance stability~**					
Herbivore population stability	0.35	0.07	4.88	0.000	0.35
Herbivore asynchrony	0.91	0.06	14.29	0.000	0.91
Herbivore richness	-0.08	0.07	-1.19	0.236	-0.08
**Herbivore population stability ~**					
Tree population stability	-0.21	0.10	-2.10	0.036	-0.21
Tree asynchrony	-0.04	0.20	-0.19	0.848	-0.04
Tree species richness	0.03	0.22	0.11	0.911	0.03
Tree FD	-0.14	0.15	-0.94	0.348	-0.14
Herbivore richness	0.68	0.14	4.93	0.000	0.68
**Herbivore asynchrony ~**					
Tree population stability	0.09	0.14	0.59	0.553	0.09
Tree asynchrony	0.27	0.24	1.13	0.258	0.27
Tree FD (Rao's Q)	-0.46	0.21	-2.20	0.028	-0.46
Herbivore richness	0.15	0.17	0.90	0.367	0.15
**Herbivore richness ~**					
Tree population stability	0.00	0.14	0.01	0.990	0.00
Tree asynchrony	0.30	0.19	1.54	0.123	0.30
Tree species richness	0.47	0.22	2.16	0.031	0.47
Tree FD (Rao's Q)	-0.27	0.15	-1.79	0.074	-0.27
**Tree population stability ~**					

Tree species richness	-0.36	0.17	-2.19	0.028	-0.36
Tree FD (Rao's Q)	0.24	0.19	1.25	0.211	0.24
**Tree FD (Rao's Q) ~**					
Tree species richness	0.74	0.07	10.72	0.000	0.74
**Tree asynchrony ~**					
Tree species richness	0.72	0.21	3.48	0.001	0.72
Tree FD (Rao's Q)	0.16	0.20	0.81	0.418	0.16

Covariances	Estimate	SE	z	P	Stand. Est.
Herbivore population stability~~					
Herbivore asynchrony	-0.15	0.10	-1.57	0.116	-0.24
Tree population stability~~					
Tree asynchrony	-0.02	0.04	-0.55	0.585	-0.04

Variances

Variable	Estimate	SE	z	P	Stand. Est.
Tree species richness	0.98	0.21	4.70	0.000	1.00
Herbivore population stability	0.49	0.10	4.96	0.000	0.50
Herbivore asynchrony	0.85	0.12	6.84	0.000	0.87
Tree FD (Rao's Q)	0.44	0.11	3.91	0.000	0.45
Herbivore abundance	0.68	0.10	6.69	0.000	0.69
Tree population stability	0.92	0.21	4.39	0.000	0.94
Tree asynchrony	0.28	0.09	3.03	0.002	0.29
Herbivore richness stability	0.49	0.07	7.47	0.000	0.50
Herbivore abundance stability	0.12	0.02	5.74	0.000	0.12

R^2

Variable	Estimate
Herbivore population stability	0.50
Herbivore asynchrony	0.14
Tree FD (Rao's Q)	0.55
Herbivore abundance	0.31
Tree population stability	0.06
Tree asynchrony	0.71
Herbivore richness stability	0.50
Herbivore abundance stability	0.88

Table S6. Path model output for tree and herbivore dynamics on generalist herbivore community stability (herbivore MPD was replaced by richness, stability measures are based on the inverse of the coefficient of variation). Statistical tests were two-sided, and no adjustments were made for multiple comparisons.

**Model**	
Estimator	Maximum likelihood
Number of observations	52
RMSEA	0.00 (90% CI: 0.00-0.13)
Chi-square	7.17
Degrees of freedom	9
P(Chi-square)/bootstrapped	0.620/0.575

Regressions

Response~Predictor	Estimate	SE	z	P	Stand. Est.
**Herbivore richness stability~**					
Herbivore population stability	0.03	0.11	0.25	0.801	0.03
Herbivore asynchrony	-0.26	0.20	-1.32	0.188	-0.26
Herbivore richness	0.29	0.12	2.33	0.020	0.29
Herbivore abundance stability	0.92	0.22	4.14	0.000	0.92
**Herbivore abundance stability~**					
Herbivore population stability	0.25	0.06	4.01	0.000	0.25
Herbivore asynchrony	0.88	0.08	11.51	0.000	0.88
Herbivore richness	0.03	0.07	0.48	0.635	0.03
**Herbivore population stability ~**					
Tree population stability	-0.25	0.09	-2.92	0.004	-0.25
Tree asynchrony	-0.05	0.21	-0.22	0.824	-0.05
Tree species richness	0.27	0.26	1.03	0.303	0.27
Tree FD	-0.33	0.15	-2.09	0.036	-0.33
Herbivore richness	0.53	0.16	3.41	0.001	0.53
**Herbivore asynchrony ~**					
Tree population stability	-0.17	0.13	-1.28	0.201	-0.17
Tree asynchrony	-0.17	0.21	-0.81	0.419	-0.17
Tree FD (Rao's Q)	0.01	0.18	0.06	0.953	0.01
Herbivore richness	-0.01	0.17	-0.04	0.969	-0.01
**Herbivore richness ~**					
Tree population stability	-0.09	0.15	-0.63	0.528	-0.09
Tree asynchrony	0.18	0.19	0.97	0.332	0.18
Tree species richness	0.61	0.21	2.91	0.004	0.61
Tree FD (Rao's Q)	-0.29	0.15	-1.94	0.048	-0.29
**Tree population stability ~**					

Tree species richness	-0.36	0.18	-2.02	0.043	-0.36
Tree FD (Rao's Q)	0.24	0.20	1.19	0.234	0.24
**Tree FD (Rao's Q) ~**					
Tree species richness	0.74	0.07	10.60	0.000	0.74
**Tree asynchrony ~**					
Tree species richness	0.72	0.21	3.48	0.000	0.72
Tree FD (Rao's Q)	0.16	0.20	0.79	0.428	0.16

Covariances	Estimate	SE	z	P	Stand. Est.
Herbivore population stability~~					
Herbivore asynchrony	0.02	0.09	0.20	0.841	0.03
Tree population stability~~					
Tree asynchrony	-0.02	0.04	-0.55	0.585	-0.04

Variances

Variable	Estimate	SE	z	P	Stand. Est.
Tree species richness	0.98	0.21	4.66	0.00	1.00
Herbivore population stability	0.49	0.09	5.25	0.000	0.50
Herbivore asynchrony	0.93	0.17	5.51	0.000	0.95
Tree FD (Rao's Q)	0.44	0.12	3.81	0.000	0.45
Herbivore abundance	0.62	0.11	5.60	0.000	0.63
Tree population stability	0.92	0.21	4.46	0.000	0.94
Tree asynchrony	0.28	0.10	2.92	0.003	0.29
Herbivore richness stability	0.32	0.06	5.06	0.000	0.32
Herbivore abundance stability	0.14	0.04	3.84	0.000	0.14

R^2

Variable	Estimate
Herbivore population stability	0.51
Herbivore asynchrony	0.05
Tree FD (Rao's Q)	0.55
Herbivore abundance	0.37
Tree population stability	0.06
Tree asynchrony	0.71
Herbivore richness stability	0.68
Herbivore abundance stability	0.86

Table S7. Path model output for tree and herbivore dynamics on specialist herbivore community stability (herbivore MPD was replaced by richness, stability measures are based on the inverse of the coefficient of variation). Statistical tests were two-sided, and no adjustments were made for multiple comparisons.

**Model**	
Estimator	Maximum likelihood
Number of observations	52
RMSEA	0.00 (90% CI: 0.00-0.07)
Chi-square	4.24
Degrees of freedom	9
P(Chi-square)/bootstrapped	0.895/0.816

Regressions

Response~Predictor	Estimate	SE	z	P	Stand. Est.
**Herbivore richness stability~**					
Herbivore population stability	0.44	0.17	2.53	0.011	0.44
Herbivore asynchrony	-0.02	0.19	-0.08	0.934	-0.02
Herbivore richness	0.24	0.14	1.64	0.100	0.24
Herbivore abundance stability	0.35	0.24	1.44	0.099	0.35
**Herbivore abundance stability~**					
Herbivore population stability	0.41	0.08	5.09	0.000	0.41
Herbivore asynchrony	0.69	0.08	8.86	0.000	0.69
Herbivore richness	0.21	0.08	2.64	0.008	0.21
**Herbivore population stability ~**					
Tree population stability	-0.15	0.14	-1.05	0.295	-0.15
Tree asynchrony	0.04	0.18	0.22	0.828	0.04
Tree species richness	-0.31	0.18	-1.68	0.094	-0.31
Tree FD	0.20	0.17	1.17	0.244	0.20
Herbivore richness	0.54	0.13	4.08	0.000	0.54
**Herbivore asynchrony ~**					
Tree population stability	0.04	0.13	0.27	0.787	0.04
Tree asynchrony	0.32	0.19	1.67	0.096	0.32
Tree FD (Rao's Q)	-0.27	0.18	-1.50	0.135	-0.27
Herbivore richness	0.39	0.13	2.95	0.003	0.39
**Herbivore richness ~**					
Tree population stability	0.13	0.14	0.94	0.346	0.13
Tree asynchrony	0.41	0.19	2.10	0.036	0.41
Tree species richness	0.21	0.23	0.89	0.374	0.21
Tree FD (Rao's Q)	-0.21	0.15	-1.44	0.151	-0.21
**Tree population stability ~**					

Tree species richness	-0.36	0.19	-1.95	0.052	-0.36
Tree FD (Rao's Q)	0.24	0.21	1.13	0.257	0.24
**Tree FD (Rao's Q) ~**					
Tree species richness	0.74	0.07	10.71	0.000	0.74
**Tree asynchrony ~**					
Tree species richness	0.72	0.21	3.47	0.001	0.72
Tree FD (Rao's Q)	0.16	0.20	0.79	0.431	0.16

Covariances	Estimate	SE	z	P	Stand. Est.
Herbivore population stability~~					
Herbivore asynchrony	-0.29	0.09	-3.12	0.002	-0.40
Tree population stability~~					
Tree asynchrony	-0.02	0.04	-0.55	0.582	-0.04

Variances

Variable	Estimate	SE	z	P	Stand. Est.
Tree species richness	0.98	0.21	4.63	0.000	1.00
Herbivore population stability	0.71	0.23	3.05	0.002	0.72
Herbivore asynchrony	0.72	0.13	5.43	0.000	0.74
Tree FD (Rao's Q)	0.44	0.12	3.69	0.000	0.45
Herbivore abundance	0.78	0.12	6.36	0.000	0.80
Tree population stability	0.92	0.22	4.20	0.000	0.94
Tree asynchrony	0.28	0.10	2.90	0.004	0.29
Herbivore richness stability	0.28	0.05	6.00	0.000	0.29
Herbivore abundance stability	0.14	0.03	4.93	0.000	0.14

R^2

Variable	Estimate
Herbivore population stability	0.28
Herbivore asynchrony	0.27
Tree FD (Rao's Q)	0.55
Herbivore abundance	0.20
Tree population stability	0.06
Tree asynchrony	0.71
Herbivore richness stability	0.72
Herbivore abundance stability	0.86

Table S8. Path model output for tree and herbivore dynamics on overall herbivore community stability (stability measures are based on the inverse of the coefficient of variation). Statistical tests were two-sided, and no adjustments were made for multiple comparisons.

**Model**	
Estimator	Maximum likelihood
Number of observations	52
RMSEA	0.00 (90% CI: 0.00-0.12)
Chi-square	8.92
Degrees of freedom	11
P(Chi-square)/bootstrapped	0.629/ 0.592

Regressions

Response~Predictor	Estimate	SE	z	P	Stand. Est.
**Herbivore richness stability~**					
Herbivore population stability	0.31	0.15	2.07	0.039	0.31
Herbivore asynchrony	-0.06	0.31	-0.19	0.846	-0.06
Herbivore MPD	0.21	0.13	1.70	0.088	0.21
Herbivore abundance stability	0.47	0.32	1.47	0.090	0.47
**Herbivore abundance stability~**					
Herbivore population stability	0.31	0.06	4.94	0.000	0.31
Herbivore asynchrony	0.91	0.06	14.59	0.000	0.91
Herbivore MPD	-0.03	0.07	-0.47	0.639	-0.03
**Herbivore population stability ~**					
Tree population stability	-0.22	0.10	-2.06	0.039	-0.22
Tree asynchrony	0.22	0.15	1.43	0.153	0.22
Tree FD	-0.19	0.14	-1.33	0.183	-0.19
Herbivore MPD	0.39	0.15	2.58	0.010	0.39
**Herbivore asynchrony ~**					
Tree population stability	0.09	0.14	0.65	0.518	0.09
Tree asynchrony	0.28	0.20	1.41	0.158	0.28
Tree FD (Rao's Q)	-0.46	0.19	-2.39	0.017	-0.46
Herbivore MPD	0.20	0.18	1.07	0.286	0.20
**Herbivore MPD ~**					
Tree population stability	-0.06	0.13	-0.48	0.635	-0.06
Tree asynchrony	0.38	0.15	2.48	0.013	0.38
Tree FD (Rao's Q)	-0.07	0.15	-0.46	0.644	-0.07
**Tree population stability ~**					
Tree species richness	-0.36	0.18	-2.01	0.044	-0.36
Tree FD (Rao's Q)	0.24	0.20	1.19	0.235	0.24
**Tree FD (Rao's Q) ~**					

Tree species richness	0.74	0.07	9.99	0.000	0.74
**Tree asynchrony ~**					
Tree species richness	0.72	0.20	3.68	0.000	0.72
Tree FD (Rao's Q)	0.16	0.19	0.87	0.386	0.16

Covariances	Estimate	SE	z	P	Stand. Est.
Herbivore population stability~~					
Herbivore asynchrony	-0.14	0.10	-1.38	0.167	-0.19
Tree population stability~~					
Tree asynchrony	-0.02	0.04	-0.55	0.586	-0.04

Variances

Variable	Estimate	SE	z	P	Stand. Est.
Tree species richness	0.98	0.20	4.83	0.000	1.00
Herbivore population stability	0.70	0.15	4.51	0.000	0.71
Herbivore asynchrony	0.83	0.13	6.50	0.000	0.85
Tree FD (Rao's Q)	0.44	0.11	3.90	0.000	0.45
Herbivore MPD	0.86	0.22	3.90	0.000	0.88
Tree population stability	0.92	0.21	4.34	0.000	0.94
Tree asynchrony	0.28	0.09	3.02	0.003	0.29
Herbivore richness stability	0.49	0.07	6.94	0.000	0.50
Herbivore abundance stability	0.12	0.02	5.96	0.000	0.12

R^2

Variable	Estimate
Herbivore population stability	0.29
Herbivore asynchrony	0.15
Tree FD (Rao's Q)	0.55
Herbivore MPD	0.12
Tree population stability	0.06
Tree asynchrony	0.71
Herbivore richness stability	0.50
Herbivore abundance stability	0.88

Table S9. Path model output for tree and herbivore dynamics on generalist community stability (stability measures are based on the inverse of the coefficient of variation). Statistical tests were two-sided, and no adjustments were made for multiple comparisons.

**Model**	
Estimator	Maximum likelihood
Number of observations	52
RMSEA	0.05 (90% CI: 0.00-0.16)
Chi-square	12.42
Degrees of freedom	11
P(Chi-square)/bootstrapped	0.333/0.387

Regressions

Response~Predictor	Estimate	SE	z	P	Stand. Est.
**Herbivore richness stability~**					
Herbivore population stability	0.17	0.10	1.714	0.09	0.17
Herbivore asynchrony	-0.31	0.23	-1.345	0.18	-0.31
Herbivore MPD	0.12	0.10	1.109	0.27	0.12
Herbivore abundance stability	0.93	0.25	3.652	0.00	0.93
**Herbivore abundance stability~**					
Herbivore population stability	0.25	0.05	5.261	0.00	0.25
Herbivore asynchrony	0.87	0.07	12.190	0.00	0.87
Herbivore MPD	0.04	0.06	0.702	0.48	0.04
**Herbivore population stability ~**					
Tree population stability	-0.31	0.11	-2.728	0.01	-0.31
Tree asynchrony	0.34	0.15	2.319	0.02	0.34
Tree FD	-0.28	0.13	-2.112	0.04	-0.28
Herbivore MPD	0.25	0.18	1.389	0.17	0.25
**Herbivore asynchrony ~**					
Tree population stability	-0.13	0.14	-0.962	0.34	-0.13
Tree asynchrony	-0.24	0.18	-1.369	0.17	-0.24
Tree FD (Rao's Q)	0.01	0.17	0.086	0.93	0.01
Herbivore MPD	0.25	0.18	1.431	0.15	0.25
**Herbivore MPD ~**					
Tree population stability	-0.16	0.13	-1.265	0.21	-0.16
Tree asynchrony	0.27	0.16	1.747	0.08	0.27
Tree FD (Rao's Q)	-0.01	0.16	-0.076	0.94	-0.01
**Tree population stability ~**					
Tree species richness	-0.36	0.19	-1.905	0.06	-0.36
Tree FD (Rao's Q)	0.24	0.20	1.220	0.22	0.24
**Tree FD (Rao's Q) ~**					

Tree species richness	0.74	0.07	10.348	0.00	0.74
**Tree asynchrony ~**					
Tree species richness	0.72	0.20	3.592	0.00	0.72
Tree FD (Rao's Q)	0.16	0.20	0.820	0.41	0.16

Covariances	Estimate	SE	z	P	Stand. Est.
Herbivore population stability~~					
Herbivore asynchrony	-0.05	0.10	-0.44	0.659	-0.06
Tree population stability~~					
Tree asynchrony	-0.02	0.04	-0.53	0.594	-0.04

Variances

Variable	Estimate	SE	z	P	Stand. Est.
Tree species richness	0.98	0.21	4.6	0.000	1.00
Herbivore population stability	0.69	0.13	5.1	0.000	0.70
Herbivore asynchrony	0.88	0.16	5.6	0.000	0.90
Tree FD (Rao's Q)	0.44	0.12	3.9	0.000	0.45
Herbivore MPD	0.88	0.21	4.1	0.000	0.89
Tree population stability	0.92	0.21	4.4	0.000	0.94
Tree asynchrony	0.28	0.09	3.0	0.003	0.29
Herbivore richness stability	0.36	0.08	4.8	0.000	0.36
Herbivore abundance stability	0.14	0.04	4.0	0.000	0.14

R^2

Variable	Estimate
Herbivore population stability	0.30
Herbivore asynchrony	0.11
Tree FD (Rao's Q)	0.55
Herbivore MPD	0.11
Tree population stability	0.06
Tree asynchrony	0.71
Herbivore richness stability	0.64
Herbivore abundance stability	0.30

Table S10. Path model output for tree and herbivore dynamics on specialist community stability (stability measures are based on the inverse of the coefficient of variation). Statistical tests were two-sided, and no adjustments were made for multiple comparisons.

**Model**	
Estimator	Maximum likelihood
Number of observations	52
RMSEA	0.00 (90% CI: 0.00-0.12)
Chi-square	8.53
Degrees of freedom	11
P(Chi-square)/bootstrapped	0.507/0.665

Regressions

Response~Predictor	Estimate	SE	z	P	Stand. Est.
**Herbivore richness stability~**					
Herbivore population stability	0.40	0.16	2.42	0.015	0.40
Herbivore asynchrony	-0.04	0.18	-0.22	0.828	-0.04
Herbivore MPD	0.30	0.13	2.32	0.021	0.30
Herbivore abundance stability	0.35	0.22	1.58	0.063	0.35
**Herbivore abundance stability~**					
Herbivore population stability	0.43	0.09	4.99	0.000	0.43
Herbivore asynchrony	0.72	0.08	9.04	0.000	0.72
Herbivore MPD	0.16	0.08	2.02	0.043	0.16
**Herbivore population stability ~**					
Tree population stability	-0.07	0.13	-0.50	0.618	-0.07
Tree asynchrony	-0.12	0.17	-0.71	0.477	-0.12
Tree FD	0.08	0.15	0.54	0.589	0.08
Herbivore MPD	0.53	0.14	3.84	0.000	0.53
**Herbivore asynchrony ~**					
Tree population stability	0.08	0.13	0.59	0.558	0.08
Tree asynchrony	0.35	0.18	1.90	0.057	0.35
Tree FD (Rao's Q)	-0.29	0.18	-1.63	0.090	-0.29
Herbivore MPD	0.36	0.14	2.49	0.013	0.36
**Herbivore MPD ~**					
Tree population stability	0.01	0.15	0.06	0.953	0.01
Tree asynchrony	0.50	0.13	3.97	0.000	0.50
Tree FD (Rao's Q)	-0.11	0.12	-0.89	0.374	-0.11
**Tree population stability ~**					
Tree species richness	-0.36	0.19	-1.91	0.057	-0.36
Tree FD (Rao's Q)	0.24	0.21	1.16	0.246	0.24
**Tree FD (Rao's Q) ~**					

Tree species richness	0.74	0.07	10.36	0.000	0.74
**Tree asynchrony ~**					
Tree species richness	0.72	0.21	3.47	0.001	0.72
Tree FD (Rao's Q)	0.16	0.20	0.80	0.427	0.16

Covariances	Estimate	SE	z	P	Stand. Est.
Herbivore population stability~~					
Herbivore asynchrony	-0.28	0.09	-3.26	0.001	-0.38
Tree population stability~~					
Tree asynchrony	-0.02	0.04	-0.53	0.600	-0.04

Variances

Variable	Estimate	SE	z	P	Stand. Est.
Tree species richness	0.98	0.21	4.57	0.000	1.00
Herbivore population stability	0.72	0.24	2.96	0.003	0.74
Herbivore asynchrony	0.74	0.13	5.92	0.000	0.76
Tree FD (Rao's Q)	0.44	0.12	3.71	0.000	0.45
Herbivore MPD	0.80	0.11	7.62	0.000	0.82
Tree population stability	0.92	0.22	4.22	0.000	0.94
Tree asynchrony	0.28	0.10	2.81	0.005	0.29
Herbivore richness stability	0.26	0.05	5.83	0.000	0.27
Herbivore abundance stability	0.15	0.03	4.96	0.000	0.15

R^2

Variable	Estimate
Herbivore population stability	0.26
Herbivore asynchrony	0.25
Tree FD (Rao's Q)	0.55
Herbivore MPD	0.18
Tree population stability	0.06
Tree asynchrony	0.71
Herbivore richness stability	0.74
Herbivore abundance stability	0.85

Table S11. Summary results of linear models for relationships between herbivore abundance stability and predictors (stability measures are based on the inverse of the coefficient of variation). Standardized parameter estimates (with standard errors, t and P values) are shown for the variables retained in the minimal models. Statistical tests were two-sided linear models, and no adjustments were made for multiple comparisons. Significance was set at $P < 0.05$.

	Abundance stability			
Overall	Est.	SE	t	P
(Intercept)	0.470	0.019	25.16	<0.001
Tree species richness	-0.056	0.023	-2.44	0.019
Tree FD (Rao's Q)	0.043	0.023	1.86	0.070
Herbivore MPD	0.027	0.024	1.12	0.267
Site B	0.026	0.028	0.91	0.370
Herbivore asynchrony	0.273	0.015	17.66	<0.001
Herbivore population stability	0.106	0.017	6.28	<0.001
Herbivore MPD: Site B	-0.062	0.029	-2.12	0.040

Generalist	Est.	SE	t	P
(Intercept)	0.563	0.019	29.21	<0.001
Herbivore asynchrony	0.321	0.019	16.49	<0.001
Herbivore population stability	0.099	0.019	5.08	<0.001

Specialist	Est.	SE	t	P
(Intercept)	0.039	0.055	0.71	0.479
Tree species richness	0.140	0.058	2.42	0.020
Tree population stability	0.053	0.026	2.01	0.050
Tree FD (Rao's Q)	-0.105	0.055	-1.90	0.064
Site B	0.123	0.080	1.55	0.130
Herbivore asynchrony	0.334	0.027	12.58	<0.001
Herbivore population stability	0.244	0.025	9.86	<0.001
Tree population stability: Site B	-0.164	0.081	-2.03	0.049
Tree FD (Rao's Q): Site B	0.142	0.075	1.88	0.066

Table S12. Summary results of linear models for relationships between herbivore richness stability and predictors (stability measures are based on the inverse of the coefficient of variation). Standardized parameter estimates (with standard errors, t and P values) are shown for the variables retained in the minimal models. Statistical tests were two-sided linear models, and no adjustments were made for multiple comparisons. Significance was set at $P < 0.05$.

	Richness stability			
Overall	Est.	SE	t	P
(Intercept)	0.959	0.026	36.59	<0.001
Herbivore MPD	0.051	0.030	1.68	0.100
Herbivore asynchrony	0.092	0.027	3.38	0.001
Tree population stability	0.112	0.030	3.74	<0.001

Generalist	Est.	SE	t	P
(Intercept)	0.978	0.030	32.15	<0.001
Herbivore asynchrony	0.161	0.031	5.24	<0.001
Herbivore population stability	0.138	0.031	4.48	<0.001

Specialist	Est.	SE	t	P
(Intercept)	0.524	0.050	10.43	<0.001
Herbivore MPD	0.203	0.066	3.09	0.003
Herbivore asynchrony	-0.051	0.079	-0.65	0.518
Herbivore asynchrony	0.095	0.045	2.09	0.042
Herbivore population stability	0.302	0.050	6.00	<0.001
Herbivore MPD: Site B	-0.145	0.078	-1.86	0.069

Table S13. Summary results of linear models for relationships between herbivore population stability and predictors (stability measures are based on the inverse of the coefficient of variation). Standardized parameter estimates (with standard errors, t and P values) are shown for the variables retained in the minimal models. Statistical tests were two-sided linear models, and no adjustments were made for multiple comparisons. Significance was set at P < 0.05.

	Herbivore population stability			
Overall	Est.	SE	t	P
(Intercept)	-0.558	0.012	-45.62	<0.001
Tree species richness	0.015	0.017	0.89	0.376
Tree population stability	-0.006	0.011	-0.52	0.606
Tree FD (Rao’s Q)	-0.027	0.014	-1.96	0.056
Site B	-0.031	0.019	-1.63	0.110
Herbivore MPD	0.028	0.010	2.95	0.005
Tree species richness: Site B	0.040	0.019	2.15	0.038
Tree population stability: Site B	-0.050	0.022	-2.33	0.025

Generalist	Est.	SE	t	P
(Intercept)	-0.541	0.012	-46.54	<0.001
Tree species richness	0.024	0.015	1.54	0.130
Tree population stability	-0.029	0.009	-3.11	0.003
Tree FD (Rao’s Q)	-0.035	0.013	-2.70	0.010
Site B	-0.051	0.018	-2.87	0.006
Herbivore MPD	0.023	0.009	2.47	0.017
Tree species richness: Site B	0.042	0.017	2.40	0.020

Specialist	Est.	SE	t	P
(Intercept)	-0.634	0.028	-22.98	<0.001
Tree population stability	0.032	0.024	1.33	0.189
Site B	0.072	0.043	1.66	0.103
Herbivore MPD	0.108	0.021	5.07	<0.001
Tree population stability: Site B	-0.117	0.048	-2.45	0.018

Table S14. Summary results of linear models for relationships between herbivore asynchrony stability and predictors (stability measures are based on the inverse of the coefficient of variation). Standardized parameter estimates (with standard errors, t and P values) are shown for the variables retained in the minimal models. Statistical tests were two-sided linear models, and no adjustments were made for multiple comparisons. Significance was set at P < 0.05.

	Herbivore asynchrony			
Overall	Est.	SE	t	P
(Intercept)	0.801	0.016	51.57	<0.001
Tree species richness	0.011	0.020	0.56	0.581
Tree FD (Rao's Q)	-0.044	0.018	-2.49	0.017
Site B	0.023	0.023	1.00	0.323
Tree species richness: Site B	0.037	0.024	1.56	0.125

Generalist	Est.	SE	t	P
(Intercept)	0.805	0.016	50.01	<0.001
Tree species richness	-0.046	0.026	-1.78	0.082
Tree population stability	-0.008	0.015	-0.51	0.613
Tree FD (Rao's Q)	0.010	0.027	0.37	0.716
Site B	0.057	0.025	2.33	0.024
Herbivore MPD	0.016	0.013	1.28	0.208
Tree species richness: Site B	0.068	0.037	1.86	0.070
Tree population stability: Site B	-0.013	0.028	-0.46	0.650
Tree FD (Rao's Q): Site B	-0.032	0.037	-0.88	0.384

Specialist	Est.	SE	t	P
(Intercept)	0.659	0.042	15.55	< 0.001
Herbivore MPD	0.143	0.043	3.35	0.002
Site B	-0.084	0.064	-1.31	0.196
Herbivore MPD: Site B	-0.101	0.065	-1.56	0.126

Table S15 Summary results of model fit statistics for the model selection procedure. Four potential models were considered during the path analysis selection procedure, each incorporating herbivore MPD, herbivore abundance, or herbivore richness, respectively. The fit statistics of the selected model are highlighted in bold. Stability measures are following Kvålseth et al[1]. (eqn. S1). Statistical tests were two-sided, and no adjustments were made for multiple comparisons.

**Herbivore MPD**	Model 1	Model 2	Model 3	**Model 4**
AIC	1119.74	1111.65	1108.63	**1107.12**
χ^2	0.00	3.91	4.89	**9.38**
p(χ^2)/ bootstrapped	NA	0.690/0.665	0.770/0.755	**0.587/0.587**
DF	0.00	6.00	8.00	**11.00**
CFI	1.00	1.00	1.00	**1.00**
SRMR	0.000	0.012	0.017	**0.021**
RMSEA	0.00(90% CI: 0.00-0.00)	0.07 (90% CI: 0.00-0.14)	0.00 (90% CI: 0.00-0.11)	**0.00 (90% CI: 0.00-0.13)**
p(RMSEA)	NA	0.752	0.829	**0.688**

**Abundance**	Model 1	Model 2	**Model 3**	Model 4
AIC	1097.87	1091.15	**1088.90**	1090.22
χ^2	0.00	5.28	**7.04**	14.35
p(χ^2)/bootstrapped	NA	0.508/0.507	**0.533/0.562**	0.214/0.307
DF	0.00	6.00	**8.00**	11.00
CFI	1.00	1.00	**1.00**	0.99
SRMR	0.000	0.017	**0.020**	0.032
RMSEA	0.00(90% CI: 0.00-0.00)	0.00 (90% CI: 0.00-0.17)	**0.00 (90% CI: 0.00-0.15)**	0.08 (90% CI: 0.00-0.17)
p(RMSEA)	NA	0.588	**0.642**	0.314

**Richness**	Model 1	Model 2	**Model 3**	Model 4
AIC	1092.34	1084.54	**1082.86**	1083.85
χ^2	0.00	4.20	**6.52**	11.51
p(χ^2)/bootstrapped	NA	0.649/0.666	**0.590/0.576**	0.425/0.385
DF	0.00	6.00	**8.00**	11.00
CFI	1.00	1.00	**1.00**	0.99
SRMR	0.000	0.014	**0.025**	0.031
RMSEA	0.00(90% CI: 0.00-0.00)	0.00 (90% CI: 0.00-0.15)	**0.00 (90% CI: 0.00-0.14)**	0.05 (90% CI: 0.00-0.17)
p(RMSEA)	NA	0.716	**0.675**	0.425

Table S16. Summary results of linear models for relationships between herbivore abundance stability and predictors (stability measures are following Kvålseth et al[1].). Standardized parameter estimates (with standard errors, t and P values) are shown for the variables retained in the minimal models. Statistical tests were two-sided linear models, and no adjustments were made for multiple comparisons. Significance was set at $P < 0.05$.

	Abundance stability			
Overall	Est.	SE	t	P
(Intercept)	0.662	0.017	39.78	<0.001
Tree species richness	-0.049	0.022	-2.26	0.029
Tree population stability	-0.023	0.015	-1.52	0.136
Tree FD (Rao’s Q)	0.039	0.021	1.88	0.067
Site B	0.008	0.027	0.31	0.760
Herbivore asynchrony	0.198	0.013	15.15	<0.001
Herbivore population stability	0.074	0.015	4.94	0.000
Tree population stability: Site B	-0.023	0.015	-1.52	0.136

Generalist	Est.	SE	t	P
(Intercept)	0.725	0.018	41.12	<0.001
Herbivore asynchrony	0.245	0.018	13.75	<0.001
Herbivore population stability	0.078	0.018	4.36	<0.001

Specialist	Est.	SE	t	P
(Intercept)	0.403	0.037	10.94	<0.001
Tree species richness	0.096	0.039	2.45	0.019
Tree population stability	0.047	0.018	2.66	0.011
Tree FD (Rao’s Q)	-0.055	0.037	-1.49	0.144
Site B	0.082	0.054	1.51	0.139
Herbivore MPD	0.045	0.024	1.84	0.072
Herbivore asynchrony	0.155	0.019	8.07	<0.001
Herbivore population stability	0.099	0.020	4.93	<0.001
Tree population stability: Site B	-0.123	0.053	-2.30	0.027
Tree FD (Rao’s Q): Site B	0.084	0.050	1.69	0.098

Table S17. Summary results of linear models for relationships between herbivore richness stability and predictors (stability measures are following Kvålseth et al[1].). Standardized parameter estimates (with standard errors, t and P values) are shown for the variables retained in the minimal models. Statistical tests were two-sided linear models, and no adjustments were made for multiple comparisons. Significance was set at $P < 0.05$.

	Richness stability			
Overall	Est.	SE	t	P
(Intercept)	0.664	0.016	40.57	<0.001
Tree species richness	-0.059	0.022	-2.73	0.009
Tree population stability	-0.026	0.015	-1.71	0.095
Tree FD (Rao's Q)	0.052	0.021	2.45	0.019
Herbivore MPD	0.017	0.021	0.82	0.417
Site B	0.008	0.026	0.32	0.754
Herbivore asynchrony	0.202	0.014	15.00	<0.001
Herbivore population stability	0.084	0.016	5.10	0.000
Tree population stability: Site B	0.059	0.031	1.91	0.063
Herbivore MPD: Site B	-0.047	0.025	-1.87	0.068

Generalist	Est.	SE	t	P
(Intercept)	1.053	0.027	39.13	<0.001
Herbivore asynchrony	0.141	0.027	5.19	<0.001
Herbivore population stability	0.121	0.027	4.45	<0.001

Specialist	Est.	SE	t	P
(Intercept)	0.022	0.030	0.73	0.471
Tree population stability	0.208	0.036	5.84	<0.001
Herbivore MPD	-0.065	0.054	-1.19	0.239
Site B	0.134	0.031	4.40	<0.001
Herbivore population stability	-0.111	0.062	-1.78	0.082
Tree population stability: Site B	-0.136	0.051	-2.65	0.011
Herbivore MPD: Site B	0.022	0.030	0.73	0.471

Table S18. Summary results of linear models for relationships between herbivore population stability and predictors (stability measures are following Kvålseth et al[1].). Standardized parameter estimates (with standard errors, t and P values) are shown for the variables retained in the minimal models. Statistical tests were two-sided linear models, and no adjustments were made for multiple comparisons. Significance was set at $P < 0.05$.

	Herbivore population stability			
Overall	Est.	SE	t	P
(Intercept)	0.141	0.003	51.72	<0.001
Tree species richness	0.006	0.004	1.59	0.119
Tree population stability	-0.002	0.003	-0.66	0.511
Tree FD (Rao's Q)	-0.007	0.003	-2.30	0.026
Site B	-0.008	0.004	-1.97	0.055
Herbivore MPD	0.006	0.002	2.92	0.006
Tree species richness: Site B	0.008	0.004	1.96	0.056
Tree population stability: Site B	-0.012	0.005	-2.48	0.017

Generalist	Est.	SE	t	P
(Intercept)	0.161	0.003	48.67	<0.001
Tree species richness	0.006	0.004	1.45	0.153
Tree population stability	-0.008	0.003	-2.98	0.005
Tree FD (Rao's Q)	-0.010	0.004	-2.69	0.010
Site B	-0.015	0.005	-2.90	0.006
Herbivore MPD	0.007	0.003	2.76	0.008
Tree species richness: Site B	0.012	0.005	2.50	0.016

Specialist	Est.	SE	t	P
(Intercept)	0.140	0.008	17.19	<0.001
Tree population stability	0.006	0.007	0.90	0.371
Site B	0.015	0.013	1.18	0.245
Herbivore MPD	0.025	0.006	4.05	<0.001
Tree population stability: Site B	-0.033	0.014	-2.32	0.025

Table S19. Summary results of linear models for relationships between herbivore asynchrony stability and predictors (stability measures are following Kvålseth et al[1].). Standardized parameter estimates (with standard errors, t and P values) are shown for the variables retained in the minimal models. Statistical tests were two-sided linear models, and no adjustments were made for multiple comparisons. Significance was set at $P < 0.05$.

	Herbivore asynchrony			
Overall	Est.	SE	t	P
(Intercept)	0.835	0.010	85.05	<0.001
Tree FD (Rao's Q)	-0.022	0.010	-2.14	0.038
Herbivore MPD	0.020	0.010	2.02	0.049

Generalist	Est.	SE	t	P
(Intercept)	0.800	0.015	52.07	<0.001
Tree species richness	-0.034	0.016	-2.11	0.040
Site B	0.069	0.023	2.98	0.004
Tree species richness: Site B	0.043	0.023	1.84	0.073

Specialist	Est.	SE	t	P
(Intercept)	0.659	0.042	15.55	<0.001
Herbivore MPD	0.143	0.043	3.35	0.002
Site B	-0.084	0.064	-1.31	0.196
Herbivore MPD: Site B	-0.101	0.065	-1.56	0.126

Table S20. Path model output for tree and herbivore dynamics on overall herbivore community stability (stability measures are following Kvålseth et al[1].). Statistical tests were two-sided, and no adjustments were made for multiple comparisons.

**Model**	
Estimator	Maximum likelihood
Number of observations	52
RMSEA	0.00 (90% CI: 0.00-0.13)
Chi-square	9.38
Degrees of freedom	11
P(Chi-square)/bootstrapped	0.587/ 0.557

Regressions

Response~Predictor	Estimate	SE	z	P	Stand. Est.
**Herbivore richness stability~**					
Herbivore population stability	0.30	0.13	2.27	0.024	0.30
Herbivore asynchrony	-0.13	0.25	-0.51	0.610	-0.13
Herbivore MPD	0.21	0.12	1.72	0.086	0.21
Herbivore abundance stability	0.58	0.23	2.48	0.013	0.58
**Herbivore abundance stability~**					
Herbivore population stability	0.28	0.07	4.05	0.000	0.28
Herbivore asynchrony	0.91	0.07	12.78	0.000	0.91
Herbivore MPD	-0.04	0.07	-0.56	0.573	-0.04
**Herbivore population stability ~**					
Tree population stability	-0.23	0.11	-2.14	0.032	-0.23
Tree asynchrony	0.26	0.17	1.55	0.120	0.26
Tree FD	-0.19	0.16	-1.23	0.218	-0.19
Herbivore MPD	0.38	0.15	2.55	0.011	0.38
**Herbivore asynchrony ~**					
Tree population stability	0.13	0.13	1.01	0.313	0.13
Tree asynchrony	0.26	0.20	1.31	0.191	0.26
Tree FD (Rao's Q)	-0.46	0.18	-2.49	0.013	-0.46
Herbivore MPD	0.22	0.18	1.22	0.221	0.22
**Herbivore MPD ~**					
Tree population stability	0.02	0.12	0.15	0.884	0.02
Tree asynchrony	0.40	0.17	2.39	0.017	0.40
Tree FD (Rao's Q)	-0.08	0.16	-0.51	0.612	-0.08
**Tree population stability ~**					
Tree species richness	-0.34	0.21	-1.66	0.096	-0.34
Tree FD (Rao's Q)	0.24	0.23	1.01	0.311	0.24
**Tree FD (Rao's Q) ~**					
Tree species richness	0.74	0.07	10.25	0.000	0.74

**Tree asynchrony ~**					
Tree species richness	0.72	0.21	3.49	0.000	0.72
Tree FD (Rao's Q)	0.16	0.20	0.81	0.419	0.16

Covariances	Estimate	SE	z	P	Stand. Est.
Herbivore population stability~~					
Herbivore asynchrony	-0.19	0.10	-2.01	0.044	-0.26
Tree population stability~~					
Tree asynchrony	-0.01	0.05	-0.18	0.860	-0.02

Variances

Variable	Estimate	SE	z	P	Stand. Est.
Tree species richness	0.98	0.21	4.74	0.000	1.00
Herbivore population stability	0.70	0.15	4.62	0.000	0.71
Herbivore asynchrony	0.82	0.11	7.18	0.000	0.83
Tree FD (Rao's Q)	0.44	0.12	3.79	0.000	0.45
Herbivore MPD	0.86	0.23	3.78	0.000	0.88
Tree population stability	0.93	0.21	4.41	0.000	0.95
Tree asynchrony	0.28	0.09	3.13	0.002	0.29
Herbivore richness stability	0.46	0.06	7.31	0.000	0.47
Herbivore abundance stability	0.16	0.03	5.39	0.000	0.16

R^2

Variable	Estimate
Herbivore population stability	0.29
Herbivore asynchrony	0.17
Tree FD (Rao's Q)	0.55
Herbivore MPD	0.12
Tree population stability	0.05
Tree asynchrony	0.71
Herbivore richness stability	0.53
Herbivore abundance stability	0.84

Table S21. Path model output for tree and herbivore dynamics on generalist community stability (stability measures are following Kvålseth et al[1].). Statistical tests were two-sided, and no adjustments were made for multiple comparisons.

**Model**	
Estimator	Maximum likelihood
Number of observations	52
RMSEA	0.04 (90% CI: 0.00-0.16)
Chi-square	12.13
Degrees of freedom	11
P(Chi-square)/bootstrapped	0.354/0.366

Regressions

Response~Predictor	Estimate	SE	z	P	Stand. Est.
**Herbivore richness stability~**					
Herbivore population stability	0.19	0.09	2.22	0.027	0.19
Herbivore asynchrony	-0.20	0.19	-1.05	0.296	-0.20
Herbivore MPD	0.10	0.10	0.94	0.345	0.10
Herbivore abundance stability	0.83	0.22	3.78	0.000	0.83
**Herbivore abundance stability~**					
Herbivore population stability	0.25	0.05	4.81	0.000	0.25
Herbivore asynchrony	0.84	0.09	9.53	0.000	0.84
Herbivore MPD	0.06	0.07	0.99	0.325	0.06
**Herbivore population stability ~**					
Tree population stability	-0.26	0.11	-2.29	0.022	-0.26
Tree asynchrony	0.33	0.15	2.26	0.024	0.33
Tree FD	-0.28	0.14	-2.04	0.041	-0.28
Herbivore MPD	0.28	0.16	1.71	0.087	0.28
**Herbivore asynchrony ~**					
Tree population stability	-0.10	0.14	-0.75	0.453	-0.10
Tree asynchrony	-0.24	0.18	-1.32	0.188	-0.24
Tree FD (Rao's Q)	0.01	0.17	0.05	0.958	0.01
Herbivore MPD	0.26	0.17	1.60	0.109	0.26
**Herbivore MPD ~**					
Tree population stability	-0.10	0.13	-0.76	0.446	-0.10
Tree asynchrony	0.29	0.15	1.89	0.059	0.29
Tree FD (Rao's Q)	-0.02	0.16	-0.14	0.888	-0.02
**Tree population stability ~**					
Tree species richness	-0.34	0.22	-1.56	0.091	-0.34
Tree FD (Rao's Q)	0.24	0.24	0.98	0.325	0.24
**Tree FD (Rao's Q) ~**					
Tree species richness	0.74	0.07	10.57	0.000	0.74

**Tree asynchrony ~**	~				
Tree species richness	0.72	0.19	3.75	0.000	0.72
Tree FD (Rao's Q)	0.16	0.19	0.86	0.389	0.16

Covariances	Estimate	SE	z	P	Stand. Est.
Herbivore population stability~~					
Herbivore asynchrony	-0.03	0.11	-0.26	0.795	-0.03
Tree population stability~~					
Tree asynchrony	-0.01	0.05	-0.19	0.851	-0.02

Variances

Variable	Estimate	SE	z	P	Stand. Est.
Tree species richness	0.98	0.21	4.69	0.000	1.00
Herbivore population stability	0.72	0.13	5.62	0.000	0.73
Herbivore asynchrony	0.88	0.16	5.46	0.000	0.90
Tree FD (Rao's Q)	0.44	0.12	3.77	0.000	0.45
Herbivore MPD	0.89	0.21	4.24	0.000	0.91
Tree population stability	0.93	0.21	4.43	0.000	0.95
Tree asynchrony	0.28	0.09	3.12	0.002	0.29
Herbivore richness stability	0.35	0.08	4.31	0.000	0.36
Herbivore abundance stability	0.18	0.05	3.70	0.000	0.18

R^2

Variable	Estimate
Herbivore population stability	0.27
Herbivore asynchrony	0.10
Tree FD (Rao's Q)	0.55
Herbivore MPD	0.09
Tree population stability	0.05
Tree asynchrony	0.71
Herbivore richness stability	0.64
Herbivore abundance stability	0.82

Table S22. Path model output for tree and herbivore dynamics on specialist community stability (stability measures are following Kvålseth et al[1].). Statistical tests were two-sided, and no adjustments were made for multiple comparisons.

**Model**	
Estimator	Maximum likelihood
Number of observations	52
RMSEA	0.07 (90% CI: 0.00-0.17)
Chi-square	13.80
Degrees of freedom	11
P(Chi-square)/bootstrapped	0.244/0.225

Regressions

Response~Predictor	Estimate	SE	z	P	Stand. Est.
**Herbivore richness stability~**					
Herbivore population stability	0.37	0.16	2.30	0.021	0.37
Herbivore asynchrony	-0.07	0.14	-0.49	0.622	-0.07
Herbivore MPD	0.35	0.15	2.43	0.015	0.35
Herbivore abundance stability	0.33	0.17	1.88	0.060	0.33
**Herbivore abundance stability~**					
Herbivore population stability	0.34	0.09	4.02	0.000	0.34
Herbivore asynchrony	0.64	0.09	7.05	0.000	0.64
Herbivore MPD	0.26	0.08	3.33	0.001	0.26
**Herbivore population stability ~**					
Tree population stability	-0.12	0.12	-0.95	0.342	-0.12
Tree asynchrony	-0.11	0.15	-0.72	0.471	-0.11
Tree FD	0.06	0.13	0.42	0.678	0.06
Herbivore MPD	0.50	0.14	3.50	0.000	0.50
**Herbivore asynchrony ~**					
Tree population stability	0.06	0.13	0.49	0.623	0.06
Tree asynchrony	0.35	0.18	1.90	0.057	0.35
Tree FD (Rao's Q)	-0.29	0.18	-1.57	0.085	-0.29
Herbivore MPD	0.35	0.15	2.39	0.017	0.35
**Herbivore MPD ~**					
Tree population stability	0.12	0.12	0.94	0.349	0.12
Tree asynchrony	0.52	0.12	4.50	0.000	0.52
Tree FD (Rao's Q)	-0.12	0.12	-1.01	0.314	-0.12
**Tree population stability ~**					
Tree species richness	-0.34	0.21	-1.67	0.096	-0.34
Tree FD (Rao's Q)	0.24	0.24	1.00	0.319	0.24
**Tree FD (Rao's Q) ~**					

Tree species richness	0.74	0.07	10.38	0.000	0.74
**Tree asynchrony ~**					
Tree species richness	0.72	0.20	3.52	0.000	0.72
Tree FD (Rao's Q)	0.16	0.21	0.79	0.432	0.16

Covariances	Estimate	SE	z	P	Stand. Est.
Herbivore population stability~~					
Herbivore asynchrony	-0.25	0.08	-3.08	0.002	-0.34
Tree population stability~~					
Tree asynchrony	-0.01	0.04	-0.19	0.849	-0.02

Variances

Variable	Estimate	SE	z	P	Stand. Est.
Tree species richness	0.98	0.21	4.64	0.000	1.00
Herbivore population stability	0.75	0.25	3.06	0.002	0.77
Herbivore asynchrony	0.74	0.13	5.87	0.000	0.76
Tree FD (Rao's Q)	0.44	0.12	3.80	0.000	0.45
Herbivore MPD	0.79	0.11	7.47	0.000	0.80
Tree population stability	0.93	0.22	4.32	0.000	0.95
Tree asynchrony	0.28	0.10	2.98	0.003	0.29
Herbivore richness stability	0.29	0.05	5.70	0.000	0.30
Herbivore abundance stability	0.21	0.05	4.53	0.000	0.21

R^2

Variable	Estimate
Herbivore population stability	0.23
Herbivore asynchrony	0.24
Tree FD (Rao's Q)	0.55
Herbivore MPD	0.20
Tree population stability	0.05
Tree asynchrony	0.71
Herbivore richness stability	0.71
Herbivore abundance stability	0.79

Table S23 Path model output for tree and herbivore dynamics on overall herbivore community stability (all herbivore individuals were included, stability measures are based on the inverse of the coefficient of variation). Statistical tests were two-sided, and no adjustments were made for multiple comparisons.

**Model**	
Estimator	Maximum likelihood
Number of observations	52
RMSEA	0.00 (90% CI: 0.00-0.06)
Chi-square	5.65
Degrees of freedom	11
P(Chi-square)/bootstrapped	0.896/ 0.890

Regressions

Response~Predictor	Estimate	SE	z	P	Stand. Est.
**Herbivore richness stability~**					
Herbivore population stability	0.27	0.22	1.25	0.210	0.27
Herbivore asynchrony	-0.17	0.48	-0.37	0.715	-0.17
Herbivore MPD	0.41	0.15	2.71	0.007	0.41
Herbivore abundance stability	0.23	0.48	0.48	0.630	0.23
**Herbivore abundance stability~**	~				
Herbivore population stability	0.39	0.03	12.51	0.000	0.39
Herbivore asynchrony	0.87	0.05	18.70	0.000	0.87
Herbivore MPD	0.05	0.03	1.55	0.122	0.05
**Herbivore population stability ~**					
Tree population stability	-0.21	0.13	-1.67	0.096	-0.21
Tree asynchrony	0.16	0.19	0.81	0.420	0.16
Tree FD	-0.21	0.19	-1.08	0.279	-0.21
Herbivore MPD	0.20	0.18	1.10	0.273	0.20
**Herbivore asynchrony ~**					
Tree population stability	0.11	0.14	0.82	0.412	0.11
Tree asynchrony	0.26	0.20	1.28	0.199	0.26
Tree FD (Rao's Q)	-0.47	0.19	-2.47	0.014	-0.47
Herbivore MPD	0.27	0.17	1.58	0.113	0.27
**Herbivore MPD ~**					
Tree population stability	-0.04	0.13	-0.27	0.786	-0.04
Tree asynchrony	0.31	0.18	1.71	0.087	0.31
Tree FD (Rao's Q)	-0.01	0.18	-0.03	0.978	-0.01
**Tree population stability ~**					
Tree species richness	-0.36	0.18	-2.01	0.045	-0.36
Tree FD (Rao's Q)	0.24	0.20	1.19	0.236	0.24
**Tree FD (Rao's Q) ~**					

Tree species richness	0.74	0.07	10.03	0.000	0.74
**Tree asynchrony ~**					
Tree species richness	0.72	0.21	3.36	0.001	0.72
Tree FD (Rao's Q)	0.16	0.21	0.77	0.439	0.16

Covariances	Estimate	SE	z	P	Stand. Est.
Herbivore population stability~~					
Herbivore asynchrony	-0.08	0.11	-0.75	0.452	-0.08
Tree population stability~~					
Tree asynchrony	-0.02	0.04	-0.52	0.606	-0.02

Variances

Variable	Estimate	SE	z	P	Stand. Est.
Tree species richness	0.98	0.21	4.79	0.000	0.98
Herbivore population stability	0.86	0.14	6.07	0.000	0.86
Herbivore asynchrony	0.80	0.12	6.70	0.000	0.80
Tree FD (Rao's Q)	0.44	0.12	3.77	0.000	0.44
Herbivore MPD	0.88	0.20	4.33	0.000	0.88
Tree population stability	0.92	0.22	4.29	0.000	0.92
Tree asynchrony	0.28	0.09	3.16	0.002	0.28
Herbivore richness stability	0.60	0.11	5.69	0.000	0.60
Herbivore abundance stability	0.05	0.01	4.15	0.000	0.05

R^2

Variable	Estimate
Herbivore population stability	0.12
Herbivore asynchrony	0.19
Tree FD (Rao's Q)	0.55
Herbivore MPD	0.10
Tree population stability	0.06
Tree asynchrony	0.71
Herbivore richness stability	0.38
Herbivore abundance stability	0.95

Table S24 Path model output for tree and herbivore dynamics on overall herbivore community stability (all herbivore individuals were included, stability measures are following Kvålseth et al[1].). Statistical tests were two-sided, and no adjustments were made for multiple comparisons.

**Model**	
Estimator	Maximum likelihood
Number of observations	52
RMSEA	0.00 (90% CI: 0.00-0.06)
Chi-square	11.19
Degrees of freedom	11
P(Chi-square)/bootstrapped	0.427/ 0.468

Regressions

Response~Predictor	Estimate	SE	z	P	Stand. Est.
**Herbivore richness stability~**					
Herbivore population stability	0.33	0.16	2.01	0.044	0.33
Herbivore asynchrony	-0.20	0.34	-0.60	0.552	-0.20
Herbivore MPD	0.30	0.14	2.21	0.027	0.30
Herbivore abundance stability	0.34	0.31	1.08	0.279	0.34
**Herbivore abundance stability~**	~				
Herbivore population stability	0.30	0.07	4.55	0.000	0.30
Herbivore asynchrony	0.89	0.07	13.19	0.000	0.89
Herbivore MPD	0.03	0.08	0.41	0.680	0.03
**Herbivore population stability ~**					
Tree population stability	-0.23	0.11	-2.05	0.040	-0.23
Tree asynchrony	0.31	0.17	1.81	0.070	0.31
Tree FD	-0.22	0.16	-1.36	0.174	-0.22
Herbivore MPD	0.31	0.15	2.13	0.033	0.31
**Herbivore asynchrony ~**					
Tree population stability	0.12	0.13	0.91	0.365	0.12
Tree asynchrony	0.26	0.20	1.29	0.197	0.26
Tree FD (Rao's Q)	-0.47	0.19	-2.45	0.014	-0.47
Herbivore MPD	0.27	0.17	1.57	0.116	0.27
**Herbivore MPD ~**					
Tree population stability	0.04	0.13	0.33	0.741	0.04
Tree asynchrony	0.33	0.18	1.84	0.065	0.33
Tree FD (Rao's Q)	-0.02	0.17	-0.10	0.924	-0.02
**Tree population stability ~**					
Tree species richness	-0.34	0.20	-1.73	0.083	-0.34
Tree FD (Rao's Q)	0.24	0.22	1.07	0.286	0.24
**Tree FD (Rao's Q) ~**					

Tree species richness	0.74	0.07	10.24	0.000	0.74
**Tree asynchrony ~**					
Tree species richness	0.72	0.21	3.48	0.001	0.72
Tree FD (Rao's Q)	0.16	0.20	0.81	0.420	0.16

Covariances	Estimate	SE	z	P	Stand. Est.
Herbivore population stability~~					
Herbivore asynchrony	-0.19	0.10	-1.89	0.058	-0.19
Tree population stability~~					
Tree asynchrony	-0.01	0.05	-0.18	0.856	-0.01

Variances

Variable	Estimate	SE	z	P	Stand. Est.
Tree species richness	0.98	0.20	4.82	0.000	0.98
Herbivore population stability	0.74	0.16	4.64	0.000	0.74
Herbivore asynchrony	0.80	0.12	6.73	0.000	0.80
Tree FD (Rao's Q)	0.44	0.12	3.81	0.000	0.44
Herbivore MPD	0.88	0.20	4.45	0.000	0.88
Tree population stability	0.93	0.21	4.39	0.000	0.93
Tree asynchrony	0.28	0.09	3.12	0.002	0.28
Herbivore richness stability	0.58	0.11	5.36	0.000	0.58
Herbivore abundance stability	0.14	0.03	5.24	0.000	0.14

R^2

Variable	Estimate
Herbivore population stability	0.25
Herbivore asynchrony	0.19
Tree FD (Rao's Q)	0.55
Herbivore MPD	0.10
Tree population stability	0.05
Tree asynchrony	0.71
Herbivore richness stability	0.41
Herbivore abundance stability	0.86

Table S25. Path model output for tree and herbivore dynamics on overall herbivore community stability (monocultures were removed, stability measures are based on the inverse of the coefficient of variation). Statistical tests were two-sided, and no adjustments were made for multiple comparisons.

**Model**	
Estimator	Maximum likelihood
Number of observations	28
RMSEA	0.07 (90% CI: 0.07-0.21)
Chi-square	12.32
Degrees of freedom	11
P(Chi-square)/bootstrapped	0.340/0.522

Regressions

Response~Predictor	Estimate	SE	z	P	Stand. Est.
**Herbivore richness stability~**					
Herbivore population stability	0.45	0.17	2.56	0.011	0.45
Herbivore asynchrony	0.44	0.44	0.99	0.321	0.44
Herbivore MPD	0.00	0.18	-0.01	0.993	0.00
Herbivore abundance stability	0.10	0.43	0.24	0.813	0.10
**Herbivore abundance stability~**					
Herbivore population stability	0.22	0.08	2.64	0.008	0.22
Herbivore asynchrony	0.90	0.09	10.38	0.000	0.90
Herbivore MPD	-0.03	0.08	-0.37	0.712	-0.03
**Herbivore population stability ~**					
Tree population stability	-0.29	0.19	-1.53	0.125	-0.29
Tree asynchrony	0.24	0.20	1.21	0.228	0.24
Tree FD	-0.33	0.19	-1.73	0.083	-0.33
Herbivore MPD	0.29	0.22	1.34	0.182	0.29
**Herbivore asynchrony ~**					
Tree population stability	0.33	0.22	1.48	0.140	0.33
Tree asynchrony	0.23	0.20	1.13	0.259	0.23
Tree FD (Rao's Q)	-0.43	0.18	-2.41	0.016	-0.43
Herbivore MPD	0.49	0.21	2.33	0.020	0.49
**Herbivore MPD ~**					
Tree population stability	-0.23	0.26	-0.87	0.382	-0.23
Tree asynchrony	0.45	0.20	2.30	0.022	0.45
Tree FD (Rao's Q)	0.00	0.20	0.00	0.998	0.00
**Tree population stability ~**					
Tree species richness	-0.36	0.24	-1.51	0.131	-0.36
Tree FD (Rao's Q)	0.27	0.20	1.38	0.166	0.27

**Tree FD (Rao's Q) ~**					
Tree species richness	0.48	0.15	3.18	0.001	0.48
**Tree asynchrony ~**					
Tree species richness	0.63	0.19	3.26	0.001	0.63
Tree FD (Rao's Q)	0.17	0.20	0.83	0.404	0.17

Covariances	Estimate	SE	z	P	Stand. Est.
Herbivore population stability~~					
Herbivore asynchrony	-0.03	0.10	-0.31	0.760	-0.05
Tree population stability~~					
Tree asynchrony	-0.04	0.08	-0.45	0.655	-0.06

Variances

Variable	Estimate	SE	z	P	Stand. Est.
Tree species richness	0.96	0.23	4.28	0.000	1.00
Herbivore population stability	0.59	0.13	4.66	0.000	0.62
Herbivore asynchrony	0.61	0.16	3.77	0.000	0.63
Tree FD (Rao's Q)	0.75	0.14	5.21	0.000	0.77
Herbivore MPD	0.69	0.15	4.73	0.000	0.72
Tree population stability	0.86	0.27	3.17	0.002	0.89
Tree asynchrony	0.45	0.14	3.35	0.001	0.47
Herbivore richness stability	0.41	0.10	4.26	0.000	0.42
Herbivore abundance stability	0.10	0.03	3.62	0.000	0.10

$\boldsymbol{R^2}$

Variable	Estimate
Herbivore population stability	0.38
Herbivore asynchrony	0.37
Tree FD (Rao's Q)	0.23
Herbivore MPD	0.28
Tree population stability	0.11
Tree asynchrony	0.53
Herbivore richness stability	0.58
Herbivore abundance stability	0.90

Table S26. Path model output for tree and herbivore dynamics on generalist herbivore community stability (monocultures were removed, stability measures are based on the inverse of the coefficient of variation). Statistical tests were two-sided, and no adjustments were made for multiple comparisons.

**Model**	
Estimator	Maximum likelihood
Number of observations	28
RMSEA	0.07 (90% CI: 0.10-0.23)
Chi-square	13.89
Degrees of freedom	11
P(Chi-square)/bootstrapped	0.239/0.499

Regressions

Response~Predictor	Estimate	SE	z	P	Stand. Est.
**Herbivore richness stability~**					
Herbivore population stability	0.26	0.17	1.49	0.097	0.26
Herbivore asynchrony	-0.15	0.46	-0.34	0.735	-0.15
Herbivore MPD	0.17	0.14	1.25	0.211	0.17
Herbivore abundance stability	0.71	0.53	1.34	0.180	0.71
**Herbivore abundance stability~**					
Herbivore population stability	0.24	0.06	4.00	0.000	0.24
Herbivore asynchrony	0.86	0.07	11.66	0.000	0.86
Herbivore MPD	0.06	0.07	0.93	0.353	0.06
**Herbivore population stability ~**					
Tree population stability	-0.32	0.21	-1.55	0.121	-0.32
Tree asynchrony	0.34	0.19	1.83	0.067	0.34
Tree FD	-0.40	0.18	-2.18	0.029	-0.40
Herbivore MPD	0.23	0.20	1.13	0.259	0.23
**Herbivore asynchrony ~**					
Tree population stability	0.09	0.33	0.29	0.773	0.09
Tree asynchrony	-0.14	0.21	-0.65	0.518	-0.14
Tree FD (Rao's Q)	0.13	0.21	0.62	0.536	0.13
Herbivore MPD	0.35	0.29	1.22	0.223	0.35
**Herbivore MPD ~**					
Tree population stability	-0.33	0.27	-1.21	0.225	-0.33
Tree asynchrony	0.35	0.20	1.74	0.082	0.35
Tree FD (Rao's Q)	0.08	0.21	0.36	0.718	0.08
**Tree population stability ~**					
Tree species richness	-0.36	0.26	-1.40	0.160	-0.36
Tree FD (Rao's Q)	0.27	0.20	1.33	0.182	0.27

**Tree FD (Rao's Q) ~**					
Tree species richness	0.48	0.14	3.43	0.001	0.48
**Tree asynchrony ~**					
Tree species richness	0.63	0.18	3.49	0.000	0.63
Tree FD (Rao's Q)	0.17	0.19	0.87	0.384	0.17

Covariances	Estimate	SE	z	P	Stand. Est.
Herbivore population stability~~					
Herbivore asynchrony	0.12	0.12	0.99	0.321	0.17
Tree population stability~~					
Tree asynchrony	-0.04	0.08	-0.49	0.624	-0.06

Variances

Variable	Estimate	SE	z	P	Stand. Est.
Tree species richness	0.96	0.24	3.97	0.000	1.00
Herbivore population stability	0.53	0.11	4.89	0.000	0.55
Herbivore asynchrony	0.86	0.19	4.64	0.000	0.89
Tree FD (Rao's Q)	0.75	0.15	4.85	0.000	0.77
Herbivore MPD	0.68	0.16	4.27	0.000	0.71
Tree population stability	0.86	0.26	3.34	0.001	0.89
Tree asynchrony	0.45	0.14	3.13	0.002	0.47
Herbivore richness stability	0.32	0.07	4.62	0.000	0.33
Herbivore abundance stability	0.09	0.02	4.21	0.000	0.09

R^2

Variable	Estimate
Herbivore population stability	0.45
Herbivore asynchrony	0.11
Tree FD (Rao's Q)	0.23
Herbivore MPD	0.29
Tree population stability	0.11
Tree asynchrony	0.53
Herbivore richness stability	0.67
Herbivore abundance stability	0.91

Table S27. Path model output for tree and herbivore dynamics on specialist herbivore community stability (monocultures were removed, stability measures are based on the inverse of the coefficient of variation). Statistical tests were two-sided, and no adjustments were made for multiple comparisons.

**Model**	
Estimator	Maximum likelihood
Number of observations	28
RMSEA	0.07 (90% CI: 0.00-0.17)
Chi-square	9.08
Degrees of freedom	11
P(Chi-square)/bootstrapped	0.615/0.743

Regressions

Response~Predictor	Estimate	SE	z	P	Stand. Est.
**Herbivore richness stability~**					
Herbivore population stability	0.07	0.15	0.47	0.642	0.07
Herbivore asynchrony	-0.62	0.26	-2.42	0.001	-0.62
Herbivore MPD	0.68	0.14	4.87	0.000	0.68
Herbivore abundance stability	0.66	0.30	2.18	0.030	0.66
**Herbivore abundance stability~**					
Herbivore population stability	0.41	0.06	6.62	0.000	0.41
Herbivore asynchrony	0.81	0.08	10.48	0.000	0.81
Herbivore MPD	0.12	0.06	1.88	0.060	0.12
**Herbivore population stability ~**					
Tree population stability	-0.13	0.20	-0.66	0.510	-0.13
Tree asynchrony	-0.09	0.26	-0.34	0.736	-0.09
Tree FD	0.10	0.23	0.42	0.671	0.10
Herbivore MPD	0.39	0.29	1.32	0.187	0.39
**Herbivore asynchrony ~**					
Tree population stability	0.15	0.18	0.85	0.393	0.15
Tree asynchrony	0.34	0.23	1.50	0.133	0.34
Tree FD (Rao's Q)	-0.30	0.20	-1.45	0.146	-0.30
Herbivore MPD	0.43	0.19	2.26	0.024	0.43
**Herbivore MPD ~**					
Tree population stability	-0.01	0.26	-0.06	0.956	-0.01
Tree asynchrony	0.56	0.14	3.85	0.000	0.56
Tree FD (Rao's Q)	-0.12	0.16	-0.77	0.441	-0.12
**Tree population stability ~**					
Tree species richness	-0.36	0.26	-1.38	0.168	-0.36
Tree FD (Rao's Q)	0.27	0.22	1.22	0.224	0.27

**Tree FD (Rao's Q) ~**					
Tree species richness	0.48	0.14	3.32	0.001	0.48
**Tree asynchrony ~**					
Tree species richness	0.63	0.20	3.15	0.002	0.63
Tree FD (Rao's Q)	0.17	0.20	0.84	0.403	0.17

Covariances	Estimate	SE	z	P	Stand. Est.
Herbivore population stability~~					
Herbivore asynchrony	-0.23	0.13	-1.71	0.088	-0.33
Tree population stability~~					
Tree asynchrony	-0.04	0.08	-0.45	0.653	-0.06

Variances

Variable	Estimate	SE	z	P	Stand. Est.
Tree species richness	0.96	0.23	4.13	0.000	1.00
Herbivore population stability	0.81	0.19	4.36	0.000	0.84
Herbivore asynchrony	0.59	0.13	4.36	0.000	0.61
Tree FD (Rao's Q)	0.75	0.15	4.87	0.000	0.77
Herbivore MPD	0.71	0.14	4.97	0.000	0.74
Tree population stability	0.86	0.27	3.13	0.002	0.89
Tree asynchrony	0.45	0.16	2.89	0.004	0.47
Herbivore richness stability	0.15	0.03	4.89	0.000	0.16
Herbivore abundance stability	0.07	0.02	3.64	0.000	0.07

R^2

Variable	Estimate
Herbivore population stability	0.16
Herbivore asynchrony	0.39
Tree FD (Rao's Q)	0.23
Herbivore MPD	0.26
Tree population stability	0.11
Tree asynchrony	0.53
Herbivore richness stability	0.84
Herbivore abundance stability	0.93

Supplementary references

1 Kvålseth, T. O. An alternative measure of ordinal association as a value-validity correction of the Goodman–Kruskal gamma. *Communications in Statistics-Theory and Methods* **46**, 10582-10593 (2017).

2 Cavender-Bares, J., Kozak, K. H., Fine, P. V. & Kembel, S. W. The merging of community ecology and phylogenetic biology. *Ecol. Lett.* **12**, 693-715, doi:10.1111/j.1461-0248.2009.01314.x (2009).
