# Supplementary material for: Asynchrony and functional diversity couple herbivore community dynamics to host plant diversity
Source: Nat Commun. 2026 Jan 15;17:762. doi: 10.1038/s41467-025-67990-0 (PMC12819501; doi:10.1038/s41467-025-67990-0)
Supplement: Supplementary file 2 — Reporting Summary [file 41467_2025_67990_MOESM2_ESM.pdf]

nature portfolio

Corresponding author(s): Ming-Qiang Wang

Last updated by author(s): Nov 4, 2025

Reporting Summary

Nature Portfolio wishes to improve the reproducibility of the work that we publish. This form provides structure for consistency and transparency in reporting. For further information on Nature Portfolio policies, see our Editorial Policies and the Editorial Policy Checklist.

Statistics

For all statistical analyses, confirm that the following items are present in the figure legend, table legend, main text, or Methods section.

n/a	Confirmed	
☐	☒	The exact sample size (*n*) for each experimental group/condition, given as a discrete number and unit of measurement
☐	☒	A statement on whether measurements were taken from distinct samples or whether the same sample was measured repeatedly
☐	☒	The statistical test(s) used AND whether they are one- or two-sided<br>*Only common tests should be described solely by name; describe more complex techniques in the Methods section.*
☐	☒	A description of all covariates tested
☐	☒	A description of any assumptions or corrections, such as tests of normality and adjustment for multiple comparisons
☐	☒	A full description of the statistical parameters including central tendency (e.g. means) or other basic estimates (e.g. regression coefficient) AND variation (e.g. standard deviation) or associated estimates of uncertainty (e.g. confidence intervals)
☐	☒	For null hypothesis testing, the test statistic (e.g. *F*, *t*, *r*) with confidence intervals, effect sizes, degrees of freedom and *P* value noted<br>*Give P values as exact values whenever suitable.*
☒	☐	For Bayesian analysis, information on the choice of priors and Markov chain Monte Carlo settings
☐	☒	For hierarchical and complex designs, identification of the appropriate level for tests and full reporting of outcomes
☐	☒	Estimates of effect sizes (e.g. Cohen's *d*, Pearson's *r*), indicating how they were calculated

Our web collection on statistics for biologists contains articles on many of the points above.

Software and code

Policy information about availability of computer code

Data collection	No software used
Data analysis	All analyses were conducted with R v 4.2.2 (packages: lme4, lavaan)

For manuscripts utilizing custom algorithms or software that are central to the research but not yet described in published literature, software must be made available to editors and reviewers. We strongly encourage code deposition in a community repository (e.g. GitHub). See the Nature Portfolio guidelines for submitting code & software for further information.

Data

Policy information about availability of data

All manuscripts must include a data availability statement. This statement should provide the following information, where applicable:

- Accession codes, unique identifiers, or web links for publicly available datasets
- A description of any restrictions on data availability
- For clinical datasets or third party data, please ensure that the statement adheres to our policy

The tree and herbivore data generated in this study have been deposited in the figshare76 at https://doi.org/10.6084/m9.figshare.30531410.v1, in the Science Data Bank77 at https://doi.org/10.57760/sciencedb.31116, in the BEF-China repository at https://data.botanik.uni-halle.de/bef-china/datasets/695. The COI sequences generated in this study have been deposited in the OMIX database of NGDC (https://ngdc.cncb.ac.cn/omix) and are available via the accession ID: OMIX013288. Source data are provided with this paper.

Research involving human participants, their data, or biological material

Policy information about studies with human participants or human data. See also policy information about sex, gender (identity/presentation), and sexual orientation and race, ethnicity and racism.

Reporting on sex and gender	N/A
Reporting on race, ethnicity, or other socially relevant groupings	N/A
Population characteristics	N/A
Recruitment	N/A
Ethics oversight	N/A

Note that full information on the approval of the study protocol must also be provided in the manuscript.

Field-specific reporting

Please select the one below that is the best fit for your research. If you are not sure, read the appropriate sections before making your selection.

☐ Life sciences ☐ Behavioural & social sciences ☒ Ecological, evolutionary & environmental sciences

For a reference copy of the document with all sections, see nature.com/documents/nr-reporting-summary-flat.pdf

Ecological, evolutionary & environmental sciences study design

All studies must disclose on these points even when the disclosure is negative.

Study description	Here, we analyze the mechanisms underlying herbivore community stability in a species-rich subtropical forest ecosystem, assessing key properties of herbivore communities and the extent to which these are coupled to the dynamics and diversity of their host communities under changing tree diversity. We used time series data (17 replicated sampling seasons of six years from 2017 to 2022 in 52 study plots) of a dominant group of insect herbivores (lepidopteran larvae) and their host tree communities in a large-scale tree biodiversity experiment (BEF-China). Specifically, we tested the relative importance of functional diversity, community asynchrony, and average population stability of both the herbivore and their host tree communities on the temporal stability of herbivore abundance and species richness.
Research sample	The study used the data on Lepidoptera herbivores and tree growth from a large-scale tree diversity experiment in forests of southeastern China.
Sampling strategy	The data were collected from 52 plots (29 plots in site A and 23 in site B), encompassing a gradient from monoculture to plots with 24 species. Specifically, per site, we considered sixteen monocultures, as well as eight 2-, four 4-, two 8-, one 16-, and one 24-species mixtures. This strategy align with the experiment design for comparability across studies.
Data collection	Lepidopteran larvae were collected for six years (from 2017 to 2022) by Ming-Qiang Wang, Yi Li, and Jing-Ting Chen. To capture seasonal changes in caterpillar assemblages and gain a rather comprehensive picture of the occurring fauna, we sampled three times per year (spring: April, summer: June and autumn: September). Caterpillars were sampled by beating individual tree branches with a padded stick and collecting all dislodged individuals that fell on a white sheet (1.5 m × 1.5 m), placed directly beneath the branches. To standardize the sampling effort, we started collecting the trees in the first row of each sampled plot and continued until 80 living trees were sampled. Because of the random planting design, this allowed us to adequately cover the complete tree species composition per plot. The aboveground wood volume of an individual tree was measured between 2016 and 2021.
Timing and spatial scale	Data for herbivores and trees were collected between 2016 and 2022.
Data exclusions	N/A
Reproducibility	All detailed description of the methods are provided, dataset could be freely avaiable online and all analyses were conducted in R. The results are fully reproducible using these data and packages.
Randomization	Apart from the number of species, tree species compositions were random. Tree compositions were randomly assigned to plots.
Blinding	N/A

Did the study involve field work? ☒ Yes ☐ No

Field work, collection and transport

Field conditions	The BEF-China tree diversity experiment is located in the subtropics of China. The experiment comprises two study sites, namely site A and site B, each spanning approximately 20 ha, and established in 2009 (site A) and 2010 (site B). The study sites are characterized by seasonal monsoon climate with a mean annual temperature of 16.7°C and a mean annual precipitation of 1800mm. The region is characterized by highly diverse native subtropical forests, predominantly composed of a blend of broadleaved evergreen and deciduous tree species.
Location	BEF-China tree diversity experiment is located at Xingangshan, Dexing, Jiangxi Province, China (29°08′–29°11′ N, 117°90′–117°93′ E).
Access & import/export	The BEF-China experiment was estabilished with permission of the local authorities in sites. All samples were fully processed in China and therefore no export permissions needed to be granted.
Disturbance	This study did not cause any environmental disturbance.

Reporting for specific materials, systems and methods

We require information from authors about some types of materials, experimental systems and methods used in many studies. Here, indicate whether each material, system or method listed is relevant to your study. If you are not sure if a list item applies to your research, read the appropriate section before selecting a response.

Materials & experimental systems

n/a	Involved in the study	
☒	☐	Antibodies
☒	☐	Eukaryotic cell lines
☒	☐	Palaeontology and archaeology
☐	☒	Animals and other organisms
☒	☐	Clinical data
☒	☐	Dual use research of concern
☐	☒	Plants

Methods

n/a	Involved in the study	
☒	☐	ChIP-seq
☒	☐	Flow cytometry
☒	☐	MRI-based neuroimaging

Animals and other research organisms

Policy information about studies involving animals; ARRIVE guidelines recommended for reporting animal research, and Sex and Gender in Research

Laboratory animals	N/A
Wild animals	The animals included in our study is lepidopteran larvae. Specifically, we collected all lepidopteran larvae individually and stored them in separate tubes filled with 99.5% ethanol. All samples were kept in a −20 ? freezer, then were used for DNA extraction for sequencing.
Reporting on sex	N/A
Field-collected samples	All samples were collected from the BEF-China experiment (with a mean annual temperature of 16.7°C and a mean annual precipitation of 1800mm).
Ethics oversight	The study did not require an ethical approval.

Note that full information on the approval of the study protocol must also be provided in the manuscript.

Dual use research of concern

Policy information about dual use research of concern

Hazards

Could the accidental, deliberate or reckless misuse of agents or technologies generated in the work, or the application of information presented in the manuscript, pose a threat to:

No	Yes	
☒	☐	Public health
☒	☐	National security
☒	☐	Crops and/or livestock
☒	☐	Ecosystems
☒	☐	Any other significant area

Experiments of concern

Does the work involve any of these experiments of concern:

No	Yes	
☒	☐	Demonstrate how to render a vaccine ineffective
☒	☐	Confer resistance to therapeutically useful antibiotics or antiviral agents
☒	☐	Enhance the virulence of a pathogen or render a nonpathogen virulent
☒	☐	Increase transmissibility of a pathogen
☒	☐	Alter the host range of a pathogen
☒	☐	Enable evasion of diagnostic/detection modalities
☒	☐	Enable the weaponization of a biological agent or toxin
☒	☐	Any other potentially harmful combination of experiments and agents

Plants

Seed stocks	The experiment was established 15 years ago and seeds were collected from local forests (see Bruelheide et al. 2014).
Novel plant genotypes	N/A
Authentication	N/A
